# Liquiñe-Ofqui’s fast slipping intra-volcanic arc crustal faulting above the subducted Chile Ridge

**DOI:** 10.1038/s41598-021-86413-w

**Published:** 2021-03-29

**Authors:** Gregory P. De Pascale, Melanie Froude, Ivanna Penna, Reginald L. Hermanns, Sergio A. Sepúlveda, Daniel Moncada, Mario Persico, Gabriel Easton, Angelo Villalobos, Francisco Gutiérrez

**Affiliations:** 1 Departmento de Geología, Facultad de Ciencias Físicas y Matemáticas (FCFM), Universidad de Chile, Plaza Ercilla 803, Santiago, Chile; 2 Department of Geography, University of Sheffield, Sheffield, S10 2TN UK; 3 Geological Survey of Norway (NGU), Postboks 6315 Sluppen, 7491 Trondheim, Norway; 4 Institute of Geoscience and Petroleum, Norwegian University of Science and Technology, Trondheim, Norway; 5 Instituto de Ciencias de la Ingeniería, Universidad de O’Higgins, Libertador Bernardo O’Higgins 611, Rancagua, Chile; 6 Department of Earth Sciences, Simon Fraser University, 8888 University Drive, Burnaby, BC V5A 1S6 Canada; 7 Golder Associates, Piso 3, Las Condes, Magdalena 181, Santiago, Chile; 8 GeoExpedition, Las Barrancas 25, Pirque, Santiago, Chile

**Keywords:** Natural hazards, Geology, Geomorphology, Seismology, Tectonics, Volcanology

## Abstract

The southernmost portion of the Liquiñe-Ofqui fault zone (LOFZ) lies within the proposed slab window which formed due to oblique subduction of the Chile Ridge in Patagonia. Mapping of paleo-surface ruptures, offsets, and lithological separations along the master fault allowed us to constrain geologic slip rates for the first time with dextral rates of 11.6–24.6 mm/year (Quaternary) and 3.6–18.9 mm/year (Late-Cenozoic) respectively. We had trouble mapping the LOFZ in one local because of a partially collapsed and previously undiscovered volcanic complex, Volcan Mate Grande (VMG: 1,280 m high and thus Vesuvius-sized) that grew in a caldera also offset along the LOFZ and has distinct geochemistry from adjacent stratovolcanoes. Besides the clear seismic and volcanic hazard implications, the structural connection along the main trace of the fast slipping LOFZ and geochemistry of VMG provides evidence for the slab window and insight into interplay between fast-slipping crustal intra-arc crustal faults and volcanoes.

## Introduction

**Figure 1:**
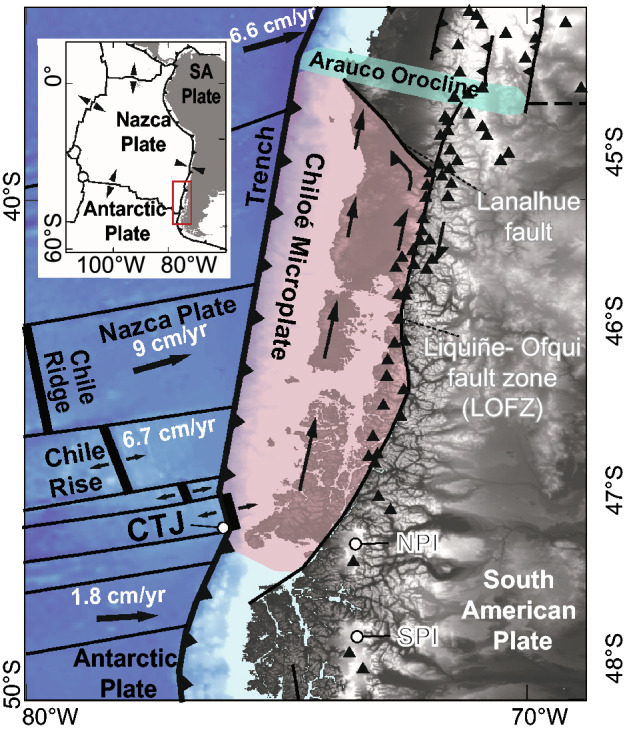
(**A**) Neotectonic and study site setting in Southern Chile and Chilean Patagonian. Master (main trace) of the LOFZ is shown. CTJ is the Chile Triple Junction or Chile Ridge/Rise between the Nazca-Antarctic-South American Plates. NPI is the Northern Patagonian Icecap (or San Valentine Icecap), SPI is the Southern Patagonian Icecap. Late Quaternary volcanoes are shown with black triangles. Oblique subduction here is the driving force for dextral motion (i.e. northwards migration of the Chiloe Microplate, after Forsythe and Nelson^10^ and Melnick et al.^21^) along the LOFZ. Base hillshade was generated with Esri ArcMap v.10.3 software (under fair terms of use, https://www.esri.com/en-us/legal/copyright-trademarks)^39^ using a digital elevation model downloaded from ALOS PALSAR Global Radar Imagery^40^ with 12.5 m resolution (https://asf.alaska.edu/data-sets/sar-data-sets/alos-palsar/).

**Figure 2:**
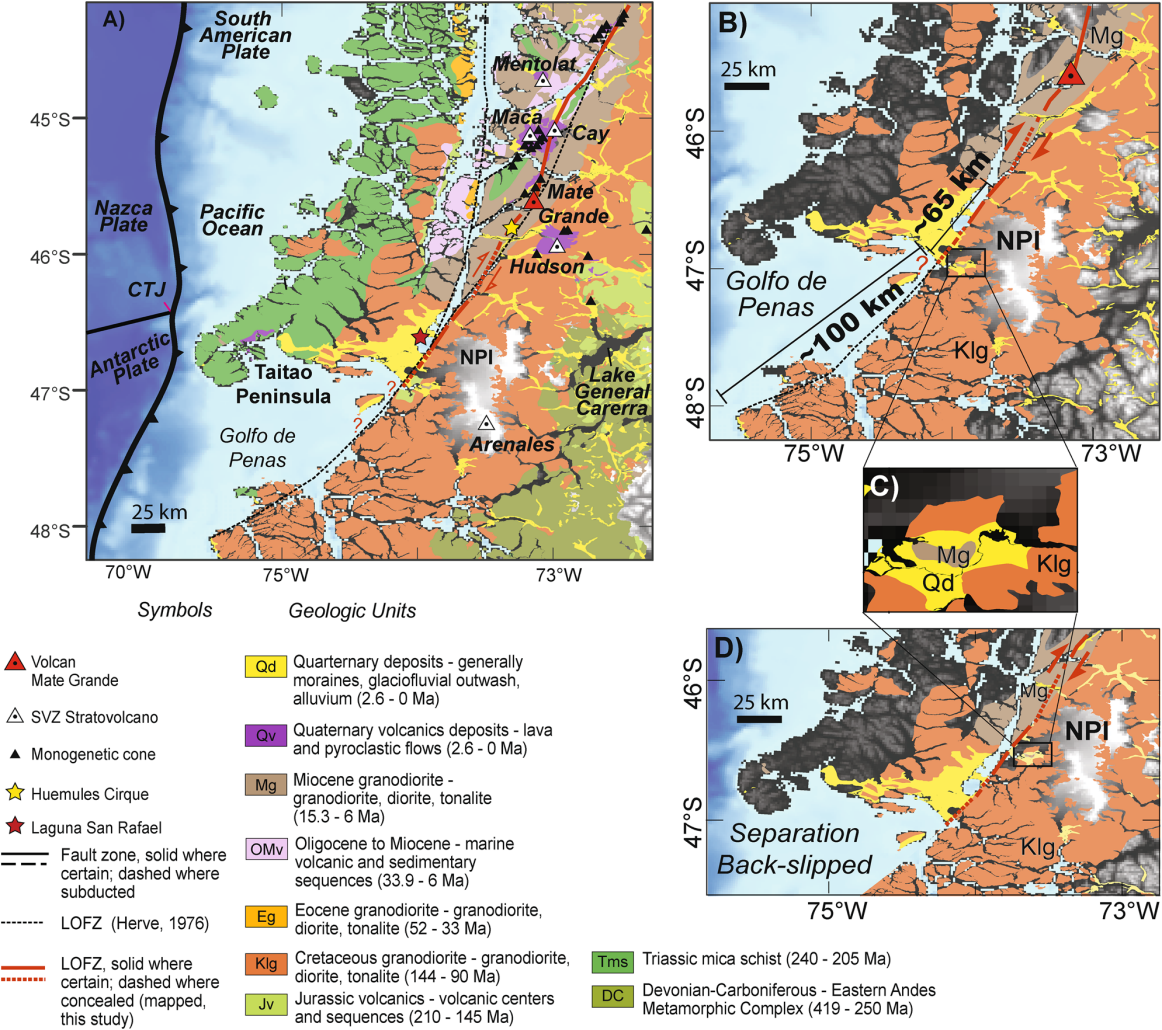
(**A**) Local setting and geology (1:1,000,000; SERNAGEOMIN, 2003) of the southernmost LOFZ, note the mapped main trace of the LOFZ, the correlation with volcanoes and LOFZ, and the location of the Volcan Mate Grande (VMG) and caldera that were discovered during this investigation. Note Hudson is the Quaternary Volcanics to the Southeast of the VMG. Note that the majority of the CM area was part of the 1960 Valdivia Mw 9.5 Giant earthquake rupture zone. (**B**) Long-term Miocene to present LOFZ indicators (the opening of the Golfo de Penas (~ 100 km) and the ~ 65 km geologic separation of the Miocene Patagonian Batholith (Mg). (**C**) Inset showing (Mg) that is located east of the LOFZ and used as a piercing point. (**D**) Retrodeformed model back-slipped by 65 km along the LOFZ to reconnect the unit Mg. Base hillshade was generated with Esri ArcMap v.10.3 software (under fair terms of use, https://www.esri.com/en-us/legal/copyright-trademarks)^39^ using a digital elevation model downloaded from ALOS PALSAR Global Radar Imagery^40^ with 12.5 m resolution (https://asf.alaska.edu/data-sets/sar-data-sets/alos-palsar/).

The Patagonian Andes are a world-class neotectonic laboratory where oblique convergence along the Southern Chilean margin subduction zone caused the largest ever measured earthquake, the 1960 M_w_ 9.5 Valdivia event^1^. Subduction of the actively spreading Chile Ridge/Rise (or Chile Triple Junction; CTJ; Fig. 1) beneath South America started during the mid-Miocene^2,3^ and is potentially a location of a slab window (e.g.^4–6^), where asthenospheric mantle can flow and mix with the upper crust due to active spreading. Seismic tomography showing clear shear wave variability supports the slab window idea^7^, however geologic data here directly above the CTJ in support of the slab window theory are limited. Because the modern convergence of ~ 66–81 mm/year^8–10^ is oblique, the crustal Chiloé Microplate (CM; or crustal sliver) is believed to be translated northwards relative to stable South America (Figs. 1, 2; e.g.^11,12^) along the dextral strike slip Liquiñe-Ofqui fault zone (LOFZ^13^). The LOFZ is one of the Earth’s major fault zones displacing continental crust and is the principal structural feature of the Main Cordillera of Chile between 38**°** S to south of the Chile Triple Junction at ~ 47**°** S near the Golfo de Penas (Figs. 1, 2^11,14^). The Golfo de Penas basin (Figs. 1, 2) developed as a pull-apart basin in response to northward movement of the CM along the LOFZ in the late Miocene^11,15^. Globally crustal faulting and seismic hazard are associated with fast-slipping (10–40 mm/year) intra-volcanic arc strike slip crustal faults, for example the at least 1200 km-long Philippine fault (20–25 mm/year^16^). Although long-term Cenozoic geologic and structural evidence suggests rapid motion along the LOFZ (e.g.^17,18^), in the 35 years since Forsythe and Nelson^11^ postulated that the Chiloe Block is moving northward relative to South America along the LOFZ due to oblique convergence or to forces in response to ridge subduction, there are still no direct measurements or field-derived estimates of strike slip slip rates. Additionally, recent thermochronological analysis from the area overlying the CTJ subduction found no evidence for reheating or thermal uplift-driven erosion and cooling within 10 to 20 Ma of the formation of the slab window^19^. Alternatively, perhaps crustal-scale faults are the main conduits for transmission of this slab window heat and fluids to the Earth’s surface if heat from the slab window is not observed in the thermochronological data. Thus, understanding how fast these faults slip could aid in understanding rates of migration of heat and fluids (including the formation of volcanoes), however there are as of yet no documented dextral Quaternary strike slip slip-rate estimates derived from geological or geomorphic evidence to confirm this activity or constrain how fast this takes place along the LOFZ. This is primarily due to the challenging access, overprinting by young volcanic and glacial cover and erosion, fiords, and dense temperate rainforest maintained by high annual rainfall (2 to 5 m/year). Importantly, there is a discrepancy between modeled rates driving the Nazca Plate, e.g. the GEODVEL model (derived from space geodesy techniques; 70.5 mm/year^20^), in contrast with geologic data that show 81 mm/year from 8.4 to 0.62 Ma^10^. Thus, geological rate data is critical to evaluate and generate both hazard and tectonic models.

Model data suggest the LOFZ is fast slipping, with geodetic rates (24 mm/year^22^), the Nuvel-1a plate motion model (28 mm/year^8^), and a limited GPS campaign (at least 6.8 mm/year^9^), all with associated uncertainties provide modeled dextral LOFZ rate estimates from ~ 6.8 to 28 mm/year, but lack field validation so far. Despite these uncertainties, the LOFZ is indeed seismically active. In 2007 a M_w_ 6.2 event in Aysén Fiord produced casualties and significant damage to infrastructure caused by landslides and landslide triggered displacement waves that field investigations combined with seismology suggest occurred along a minor strike-slip fault within the LOFZ^23^. Furthermore, microseismicity along the Southern LOFZ demonstrates its active nature^24^. Kanamori and Rivera^25^ reevaluated source characteristics of a June 6, 1960 M_s_ 6.9 earthquake within the 1960 Valdivia aftershock sequence, where they revised this event to instead a shallow dextral M_w_ 7.7 event along the LOFZ (although no surface rupture was ever documented). Analysis of submarine data after the 2007 event suggested that the main trace of the LOFZ did not rupture to the seafloor (which they refer to as the “Rio Cuervo fault”), and with subtle evidence, which in places is obscured by submarine landslides, to suggest instead that seafloor rupturing occurred along a minor fault parallel to the main LOFZ^26^. Because slip rates provides a key parameter to how fast a fault system is loaded and thus can provide insight into timing between major earthquakes, geological characterisation can help with hazard models as well.

**Figure 3:**
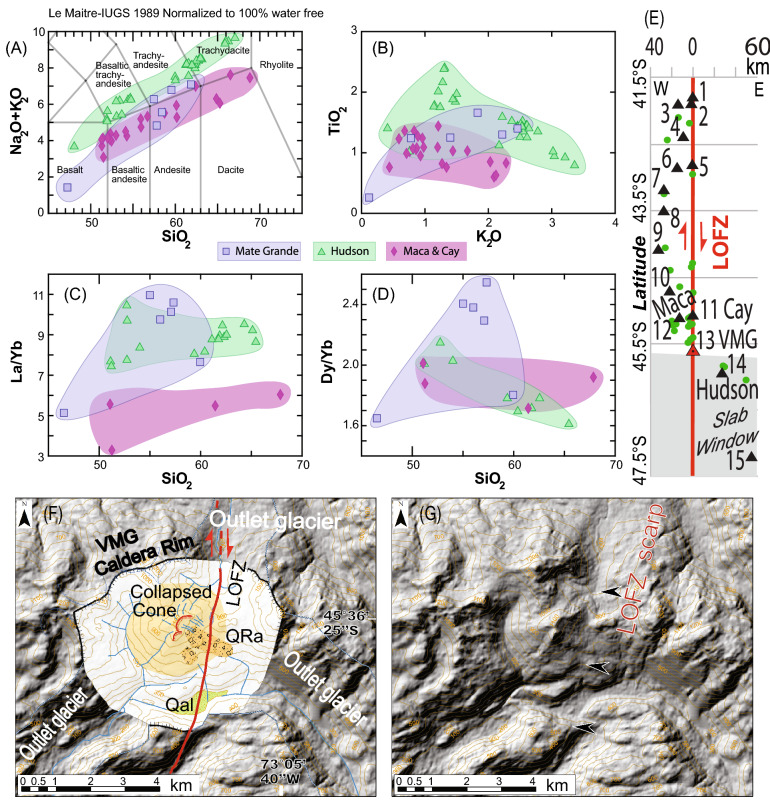
(**A**) Volcan Mate Grande (VMG: discovered during this study; 45° 35′ 28″ S, 73° 07′ 51″ W) has Hi-K calcalckaline magmas and (**B**–**D**) distinct geochemical characteristics compared with Volcan Maca and Cay complexes (*32, 33*; VMCC: typical SVZ magmas) and Volcan Hudson (VH; atypical SVZ magmas). VMG has distinctive La/Yb (**C**) and Dy/Yb (**D**) values, i.e. a completely different signature between VMG magmas and VH magmas. (**E**) Distribution (perpendicular distance (km) and normalised along-strike of the LOFZ) of stratovolcanoes and monogenetic cones from 41.5° to 47.5° S based on our mapping. (**F**,**G**) DEMs and mapping from the VMG, showing the 5 km by 4 km caldera, the young partially collapsed cone, the rock avalanche deposits (QRa), Quaternary alluvium (Qal), and the location of the main trace of the LOFZ that cuts this cone and displaces the rock avalanche deposit (likely triggered by a LOFZ earthquake/rupture) northward (i.e. dextrally) by ~ 170 ± 20 m. Base hillshade was generated with Esri ArcMap v.10.3 software (under fair terms of use, https://www.esri.com/en-us/legal/copyright-trademarks)^39^ using a digital elevation model downloaded from ALOS PALSAR Global Radar Imagery^40^ with 12.5 m resolution (https://asf.alaska.edu/data-sets/sar-data-sets/alos-palsar/).

The bedrock geology along the area above the subducted CTJ (Fig. 2) is dominated by the Jurassic to Miocene intrusive Patagonian Batholith (e.g.^27,28^) and Southern Volcanic Zone (SVZ). There are at least 30 Holocene-active SVZ volcanoes located within or near the southernmost 400 km of the LOFZ (Figs. 1, 2, 3^29^). This area was completely covered by the Patagonian Ice Sheet (with two major icecaps persisting in the area until today, i.e. Figs. 1, 2) during the Last Glacial Maximum (LGM^30^) at 27–25 ka with initial deglaciation after 18–17 ka, and the Younger Dryas glacial advance from 12.9 to 11.7 ka^31^. This study aims to obtain first order field-derived Late-Cenozoic and Late-Quaternary strike slip, dextral slip rates along the master fault of the LOFZ through a combination of remote sensing and field observations to better understand the neotectonics in Patagonia and associated geohazards.

## Methods

We reviewed the literature, geological maps and then mapped the main traces of the LOFZ using SPOT imagery (20 m resolution) in combination with Google Earth. During remote sensing, two main targets were discovered, the Huemules Range and Cirque (HC) where the LOFZ appears to offset a glacial valley and cirque in addition to offsets near the Laguna San Rafael (Fig. 2), which were then visited in the field (in 2015, 2016, 2017 and 2018) using a combination of boat travel in the fiords and by helicopter. Importantly, we identified young geomorphic surfaces (e.g. lahar plains, rivers) to clearly outline where erosion or deposition inhibits our ability to see the trace of the fault (i.e. where not to look), and focused on older landforms that would preserve longer-term slip accumulation in the landscape.

A combination of satellite imagery (Google Earth, 20 m SPOT imagery and Planet Labs), and a digital elevation models (DEM) derived from a 30 m ALOS PALSAR (i.e. the Phased Array type L-band Synthetic Aperture Radar, which is an satellite-based active microwave sensor using L-band frequency), helicopter and boat reconnaissance with field photography and observations from fieldwork were used to map the main trace of the LOFZ above the CTJ. In this study, the “main trace” is defined in a Late-Quaternary neotectonic sense, as the principle Holocene LOFZ trace (i.e. the master fault, and not minor splays or subsidiary faults) with geomorphic expression (i.e. clear post-LGM traces) and by not exhumed ductile zone fault rocks that perhaps have older deformation histories that are not representative of contemporary brittle-zone (i.e. upper crustal) deformation along the LOFZ. We focused our mapping specifically on compiled regional geology (for Late-Cenozoic bedrock lithological separations, Fig. 2) and on short-term Late-Quaternary tectonic geomorphology for geomorphic offsets. The fault trace mapping (e.g.^32^) focused on mapping the main trace of the fault based on fault scarps and was identified based on generally north–south lineaments cutting topography, bedrock and Quaternary deposits, deflecting rivers, and expressed as linear valleys (even under dense vegetation cover) with associated vegetation lineaments. Stratovolcanoes and monogenetic cones were mapped from the regional geology and in Google Earth and their distribution versus perpendicular distance from our mapped trace of the LOFZ were plotted (Fig. 3, see Supplemental Data). Remote mapping was validated with fieldwork and field sampling by helicopter, boat, and on foot. Slip rates were calculated by converting measured distances (including uncertainties which were associated by best fit matching and use of retro-displacements) of along-fault geomorphic displacements, or geologic separations to millimeters, and then dividing these distances by lithologic or geomorphic landform ages (Table 1, see Supplemental Data). We used structure from motion (SfM) photogrammetry techniques to map the HC area by collecting sets of overlapping areal photographs using a DSLR camera with overflights by helicopter over the Huemules Range. These photographs were then processed using Argisoft Photoscan Pro software and a 3D model (that can be viewed as a 3D pdf in Adobe Acrobat) was then developed from these data (please see Supplemental Data).

**Figure 4:**
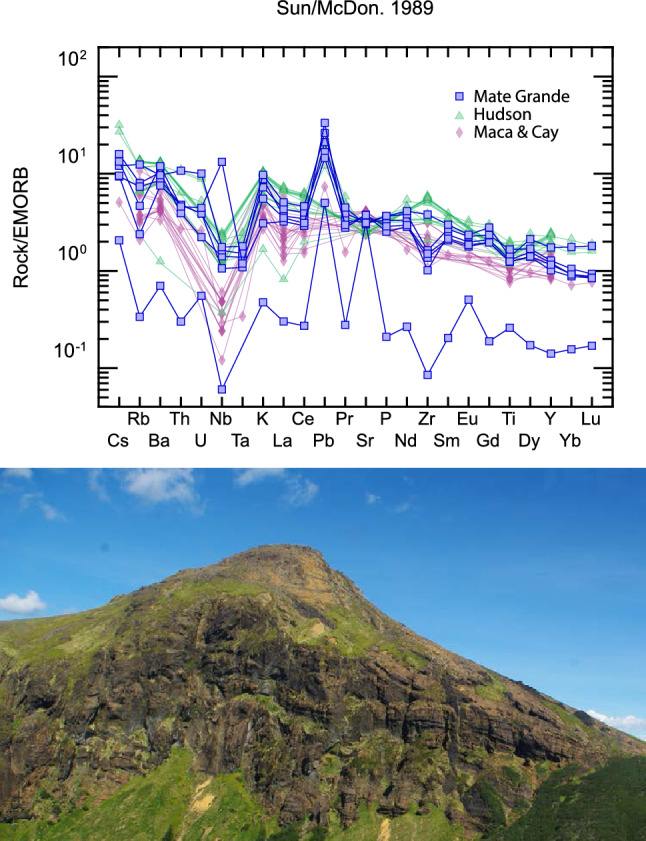
At top is an enriched Morb normalized spider diagram with samples from VMG. Intermediate samples with MgO contents between 2 and 4 of Hudson, Macá and Cay volcanoes were selected for comparison between the volcanoes. Note the distinct signature of the VMG versus the nearby other major stratovolcanoes^33,34^. At bottom is an oblique helicopter photo taken in January 2016 of the VMG during field reconnaissance. Please note the mafic nature of the stratified volcanic rocks here (consistent with the spider plot above) and flow bands that formed during VMG eruptions. Field photograph by R. Hermanns. Please see Supplemental Information for additional field photographs.

The fiord immediately south of Volcan Mate Grande (VMG; Figs. 1, 2—please see Supplemental Data) was visited during the CIMAR24 marine and coastal investigation in 2018 in the Fiords of Southern Chile and sampling of the volcanic materials (e.g. lavas and tuffs, which are more mafic and thus in stark contrast with the dominantly felsic Patagonian Batholith here) from this volcano were taken from small streams (< 3 m wide channels) that descend from the flanks of VMG (< 4 km from VMG to the Fiord here) and with clear catchments that are not connected to other nearby volcanic centres (e.g. Hudson). The geochemical samples for the VMG were processed by ALS Patagonia S.A. in Santiago, Chile including a 4 acid digestion multi-element ICP-MS with rare earth elements and XRF analysis. These data were then compared with geochemical data from nearby Volcanic centres (Figs. 1, 2, 3, 4^33,34^).

### Fast slipping LOFZ and Volcan (and Caldera) Mate Grande

**Figure 5:**
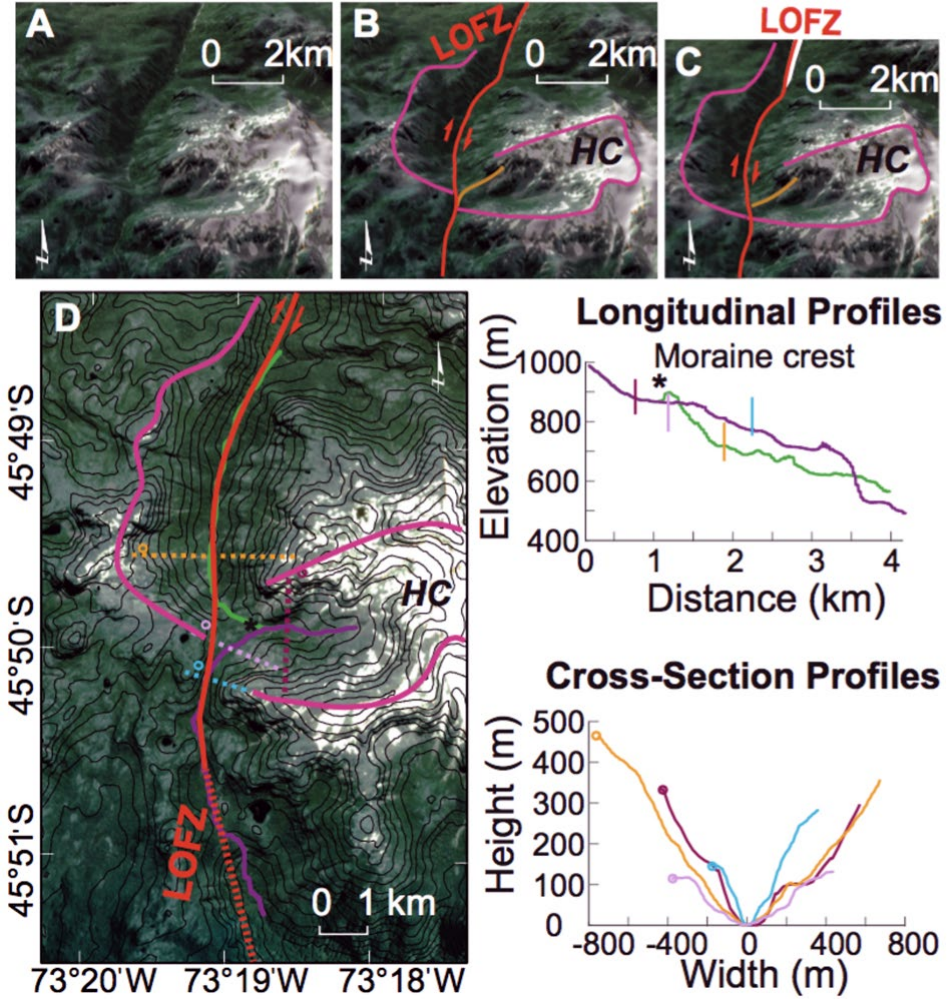
(**A**) Huemules Cirque (HC) setting based on ALOS PALSAR 30-m imagery. (**B**) Key features including the LOFZ and the outline of valley margin. (**C**) Retrodeformed model with the valley margin back-slipped along the LOFZ by 400 m which realigns the glacial valley to when it was last fully occupied by ice during the LGM (at least 17.3 ka). (**D**) Map showing locations of key elements and topographic profiles. (**E**) Topographic profiles. Note that the yellow and orange profiles provide a profile of the glacial Humules Valley and are now offset along the LOFZ have an excellent fit. Thus rapid LOFZ dextral motion (i.e. 21.7 to 24.6 mm/year) is causing the Huemules Valley to be almost beheaded from the HC. Field and helicopter photos from this site are shown in Fig. 6. Base hillshade was generated with Esri ArcMap v.10.3 software (under fair terms of use, https://www.esri.com/en-us/legal/copyright-trademarks)^39^ using a digital elevation model downloaded from ALOSPALSAR Global Radar Imagery^40^ with 12.5 m resolution (https://asf.alaska.edu/data-sets/sar-data-sets/alos-palsar/).

**Figure 6:**
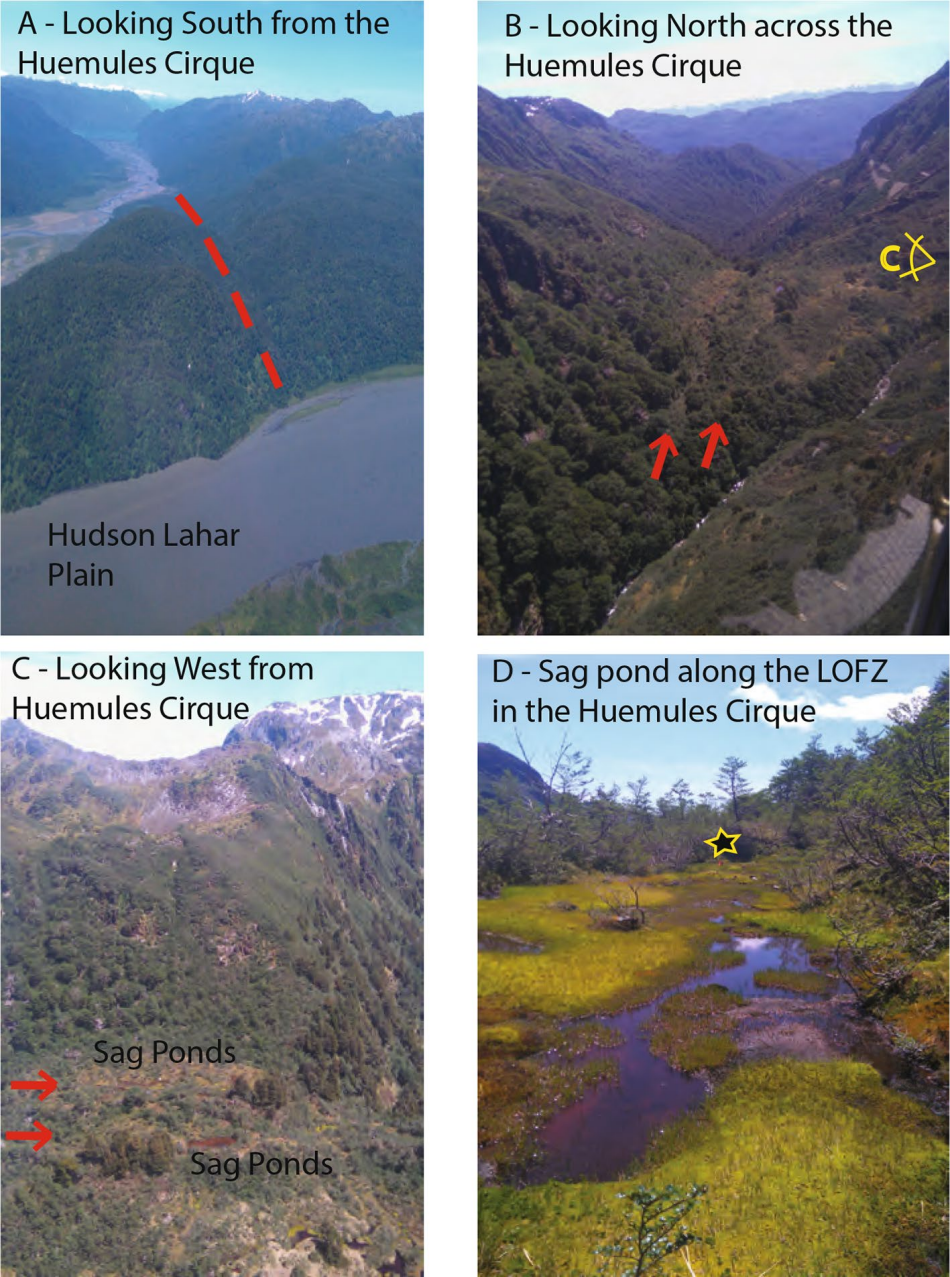
Field photos from January 2016 from along the master fault within the LOFZ in and around the Huemules Cirque (HC). (**A**) Oblique helicopter photo looking south toward Exploradores Bay and Laguna San Rafael from the HC. Main fault trace is the low forested valley. Note the young lahar plain from the Hudson Volcano. (**B**) Oblique helicopter photo looking north along the LOFZ over the HC. Red arrows show two lineaments consistent with displacements observed (shown in Fig. 5), and sag ponds. Note the widening of the main HC valley to the North versus the narrow valley with the Huemules Creek draining south. Orientation of photo C shown in panel. (**C**) Oblique Helicopter photo looking West (position shown in (**B**)), showing the two main lineaments along the master LOFZ and sag ponds. Note how the valley widens on the right of the photo as per the width of the main glacial valley that is being translated northwards by long term dextral slip along the LOFZ (i.e. the HC is being beheaded over time due to northwards dextral motion along the LOFZ). (**D**) Field photograph looking North along the LOFZ at the sag pond shown in (**C**). This low-lying (along strike) linear pond is consistent with the main damage zone of the master fault here. Star is located above a machete with flagging tape attached stuck in the ground for scale (~ 40 cm is above ground in photo). Field and Helicopter Photographs by G.P. De Pascale.

Due to Holocene erosion, the presence of Late-Quaternary volcanic cover, and numerous fiords, only ~ 25% of the main trace (i.e. fault scarps formed along the master fault) of the LOFZ can be clearly mapped along the southernmost 400 km (Figs. 1 and 2, Supplementary Info). In the terrestrial sections (from Golfo de Penas to 44° S, Fig. 1) the master Liquiñe-Ofqui fault has a north-northeast trend with typical dextral-reverse (up to the East) strike-slip tectonic geomorphology. Here we present a detailed description of our observations along this 400-km-long section of the master Liquiñe-Ofqui fault (Fig. 2; please also see Supplemental Materials). Along the the southern end of the Golfo de Pena, the LOFZ is speculated to exist although never indisputably presented based on field mapping and marine geophysical data running along the southern margin of the Golfo de Pena and the coast for 140 km where it emerges onshore at the Ofqui Peninsula and forms the boundary between the high Patagonian Andes to the east and low-lying Quaternary glacial deposits and lower-lying mountainous terrain to the west^10,12^. From a geomorphic perspective (Fig. 2), the LOFZ surface trace is clear, strikes northeast (028 azimuth), straight and easily detected from the Ofqui Peninsula north for 81 km (except where it submarine, which is never > 4 km long; Fig. S1) where it enters a major fiord/bay at the Bahia Exploradores. While 66 km north of the Golf de Pena, a secondary trace of splays off of main the LOFZ and trends northwest (355 azimuth) into the fiords. The main trace of the LOFZ is inferred to continue along the same trend (024 azimuth) from Bahia Exploradores north because 48.7 km north of where it goes offshore the trace emerges onshore where it transects Patagonian Batholith bedrock terrain for another 2.7 km with clear dextral displacements before it again is obscured by the 2.5 km wide Rio Huemules lahar plain (which decends from Volcan Hudson), and then trends north into the Patagonian Batholith-composed Huemules range and cirque (HC). There a 2.4 km^2^ Late Quaternary glacial cirque is cut off from it's valley due to dextral motion (i.e. a number of displacements during fault ruptures over various seismic cycles) along the LOFZ (with the cirque valley wall displaced ~ 400 m, Figs. 5, 6). From the southern edge of the displaced HC the master fault scarp trace is clear for 18.5 km, cutting across topography with a number of clear stream deflections, before it heads underwater at the Quitralco Fiord near the VMG. North of the Quitralco Fiord presents a bit of an enigma as the master fault cannot be easily seen in remote sensing data (i.e. SPOT imagery or Google Earth) to cut across the mapped Patagonian Batholith for the ~ 28 km section from Quitralco to Aysen Fiord and presents a stark clear contrast from a clear strike-slip trace that cuts across topography with abundant evidence for dextral displacements immediately to the south. This area is coincident with the VMG that we found during our Helicopter reconnaissance along the LOFZ (and later sampled during fieldwork in Quitralco Fiord). Retrospectively (post discovery during our helicopter flight) a review of ALOS PALSAR 12.5 m DEM revealed that the VMG is found within a much larger caldera and VMG's youngest cone is cut in half due to a collapsed flank of this stratovolcano (Figs. 3, 4). The Quaternary volcanic landslide debris (i.e. QRa - Quaternary rock avalanche) is displaced dextrally (i.e. western side of the master fault is translated northwards) by the main trace of the LOFZ and provides insight into interplay between volcanism, landsliding, and active tectonics (Fig. 3). North of VMG, the main LOFZ trace trends northwards to Aysen Fiord, cutting across the fiord and emerges onshore east of the Volcan Maca and through Volcan Cay where it again trends north into the fiord of the Puyuhaipue Canal (Figs. 2, 3).

The oldest potential indicator of long-term Late-Cenozoic LOFZ dextral strike slip motion is from the northward movement (~ 100 km) of the CM along the LOFZ, and subsequent extension at its southern end since the Late Miocene (11.6 Ma), which caused a major gap in the coastline, the Golfo de Penas (Figs. 1, 2^11,12,15^). If this opening of the Golfo de Penas was not only dextral, but also due to tectonic subsidence (i.e.^10^), then the horizontal separation would be smaller than 100 km. From the Ofqui Peninsula northward, to the east of the LOFZ, the bedrock is mostly Early Cretaceous Northern Patagonia Batholith (Klg; 144–90 Ma), but immediately south of the Ofqui Peninsula near the Golfo de Penas, the younger Miocene igneous intrusive rocks (the Northern Patagonia Batholith—Mg, 18–5.3 Ma^28^) crop out (Fig. 2). If, as mapped, this same Miocene rock unit is found ~ 65 km north of the Golfo de Penas on the west side of the LOFZ, it is a measurable geologic separation and provides a piercing point to constrain long-term horizontal dextral fault motion (Fig. 2, Table 1). If we assume these plutons were emplaced simultaneously along the LOFZ^14^ and then separated due to LOFZ motion, a minimum dextral LOFZ separation of 65 ± 5 km can be inferred (Fig. 2). Future dating, petrographic description, or detailed mapping could prove that these are instead distinct intrusive bodies with similar timing. Nevertheless, as this is a minimum, horizontal separations closer to those from the opening of the Golfo de Penas (~ 100 km over 10 Ma^12^) are possible (Fig. 2). Thus, geologic separations on the order of 65 km and up to a maximum of ~ 100 km (Fig. 2) provides a Miocene (18 Ma) to present total slip estimates along the LOFZ in the Late-Cenozoic, with corresponding first order dextral strike slip rates estimates ranging from 3.6 to 18.9 mm/year over longer time scales than the more-reliable Late-Quaternary rates discussed above (Table 1).

**Table 1:** Late-Cenozoic to present LOFZ geological seperation and displacement evidence with associated dextral, strike slip, slip rates.

Number, type of displacement and description	Displacement (km or m)	Age range (years)	Study evidence is derived from	Slip rate or range (mm/year)
(1) Geological and Geomorphic Offset, Golfo de Pena, the roughly circular embayment that penetrates approximately 100 km into the Southern Andes and which likely formed as a pull-apart basin in response to the northward migration of the Chiloe Block along the LOFZ. Provides a minimum slip rate since 11.6 to 5.3 Ma (Figs. 1 and 2)	~ 100 km	11.6 to 5.3 Ma Late Miocene	Forsythe and Nelson (1985), Murdie et al. (1993)	8.6 to 18.9 (mean 13.8)
(2) Geologic Separation; Miocene Granodiorites, Diorites and tonalities, located East of the LOFZ near the northern end of the Golfo de Penas (Shown in Fig. 2). West of the LOFZ, this same unit is located -65 km to the north. Provides a minimum slip rate since 18 to 5.3 Ma (Fig. 2)	65 ± 5 km	18.0 to 5.3 Ma Miocene	This study using *SERNAGEOMIN, 2003* regional geology	3.6 to 12.3 (mean 8.0)
(3) Quaternary geomorphic offset, Huemules Range, with an offset southern valley wall from the Huemules Cirque (HC). Provides a post-LGM dextral slip rate (Figs. 5 and 6)	~ 400 ± 25 m	17.3 ka Post-LGM Quaternary	This study—remote sensing & SfM. Deglaciation not earlier than 18–17 ka from Hein et al. (2010)	21.7 to 24.6 (mean 23.1)
(4) Quaternary geomorphic offset, Huemules Range, offset of a cliff wall, likely post-Younger Dryas (YD). (Figs. 5 and 6)	150 m	12.9 to 11.7 ka Post-Younger Dryas	This study using remote sensing SfM. Younger Dryas was from 12.9 to 11.7 ka from Glasser et al. (2012)	11.6 to 12.8 (mean 12.2)
			Arithmetic mean dextral late-cenozoic LOFZ strike slip	14.3 ± 7

The main trace of the LOFZ is obscured by a previously undocumented major northeast-striking Quaternary 1280-m-high volcanic ridge, caldera, and young stratovolcano that we discovered during helicopter reconnaissance (Volcán Mate Grande, VMG, 45° 35′ 28″ S, 73° 07′ 51″ W—in a place that we had trouble mapping the fault during remote sensing). The caldera (Fig. 3) is oval in shape and measures ~ 5 km by 4 km in diameter, and its walls were partially eroded by three outlet glaciers (which were confined to the valleys as opposed to LGM ice which in this area covered everything) associated with a Late Quaternary ice cap (likely only Younger Dryas in age 12.9 to 11.7 ka because caldera walls are otherwise intact^31^). The VMG's youngest cone is composed of mafic lavas and has a semi-circular morphology along the base within the caldera with a radial drainage pattern. As this youngest cone is found in the middle of the caldera and has no evidence of glacial erosion, it strongly suggests genesis in the Holocene (Fig. 3). Growing inside the caldera, the summit of this volcanic cone has only one half of the crater preserved, the cone experienced a partial collapse of the Southeastern flank generating a rock avalanche which left only the northwestern portion of the original crater intact at the summit (Fig. 3). Part of the rock avalanche deposit is displaced northwards (dextrally) along the main LOFZ, which cuts across the VMG and clearly visible with a fault scarp, by 170 ± 20 m, thus with at least ~ 170 m of Holocene slip, which suggests that considerable slip has taken places along the main trace of the LOFZ since this collapse took place. Given the location of the VMG along the LOFZ, it is plausible a LOFZ surface rupture/earthquake triggered the collapse.

Geochemistry from VMG samples (Figs. 3, 4) are distinct from adjacent SVZ stratovolcanoes including Hudson which is the first volcano within the SVZ found east of and off of the main trace of the LOFZ (Figs. 1, 2^33^), and Maca and Cay^34^ which are found further north. VMG intermediate magmas have major element values between Hudson and Maca and Cay Volcanoes, reaching TiO2 and K2O values as high as Hudson (Fig. 3). However, VMG has high La/Yb values, similar to Hudson and very high Dy/Yb ratios, suggesting a unique distinctive signature possibly attributed to a particular melting condition. The most mafic sample of VMG has high MgO and very low incompatible elements, and is one of the most primitive volcanic samples found in modern volcanoes in this region, including monogenetic cones (Fig. 4^33,34^). Despite all these particular geochemical signatures, VMG has a calc-alkaline signature (e.g. low Nb and high Pb) related to subduction zones (Fig. 4).

Thirty kilometers to the south-west of the VMG, the Huemules Cirque (HC) and glacial valley is dextrally offset by at least 400 m along the master LOFZ (Figs. 2, 5, 6, Table 1). Along the main trace here the fault scarp has sag ponds and coincident expressive vegetation lineaments (Fig. 6). The Southern edge of this glacial valley is bisected by the LOFZ, translating the block to the west of the LOFZ northward and almost blocking the valley exit. This northward displacement resulted in the valley (which previously drained to the north) being cut-off (i.e. nearly beheaded) from the source cirque, and the development of a new drainage route to the south (Figs. 5, 6). The topographic profiles of valley geometry upstream and downstream of the LOFZ displacement both contain a post-LGM lateral moraine at the same relative elevation to the valley thalweg, which demonstrates the misfit of this currently configuration (Figs. 5, 6). This suggests that the geomorphic offset and misfit valleys formed since the location was fully glaciated during the LGM (17.3 ka^29^) and was likely ice free by 11.5 ka^30^; (Figs. 2, 5) due to northward dextral displacement along the main trace of the LOFZ. A post-LGM lateral moraine, evident in the north drainage longitudinal profile (Fig. 5), indicates stream piracy of the HC glacier which was captured and now to flows to the south. The valley geometry of the south drainage at the knickpoint is v-shaped and does not contain the developed lateral moraine of the northward drainage (Fig. 5), suggesting the youngest HC glacier (i.e. post-LGM) most likely terminated upstream of this knickpoint. The offset of bare rock ridges denoting the HC catchment boundary in this southern drainage (Fig. 5) are measured in the SfM model (Supplementary Data); at this location the displacement is ~ 150 m. Late-Quaternary, valley-scale geomorphic displacements demonstrate that the southernmost master fault within the LOFZ is fast slipping from 11.6 to 24.6 mm/year with an arithmetic mean of 14.3 ± 7 mm/year (Table 1).

### Fast slipping LOFZ, association with volcanism, and slab window implications

Our investigation shows that above the subducted CTJ the main trace of the LOFZ is localised (i.e. there is a clear, narrow, < 100 m wide, and oftentimes much narrower on the order of 10’s of meters, and easily visible in remote sensing data) fault scarp which is the fastest-slipping crustal strike slip fault in the Patagonian Andes (~ 700 km to the south of the LOFZ the plate boundary Magallanes fault has a geologic sinistral slip rate of 7.8 ± 1.3 mm/year^35^. Our Late-Quaternary slip rates for the master fault here (11.6 to 24.6 mm/year) provide the first field validation for geodetic (24 mm/year^22^), the Nuvel-1a (28 mm/year^8^), and GPS campaign (~ 6.8 mm/year^9^) derived models. Our results also support the Kanamori and Rivera^25^ M_w_ 7.7 crustal strike slip dextral event in 1960 with clear long-term dextral displacements consistent with surface faulting as would be expected after a dextral strike slip surface rupture. This is important because until our study, no clear evidence of terrestrial LOFZ surface faulting was reported to support this idea, although submarine evidence suggest repeated events within the past 12 ka^26^. Perhaps then, the Chile Margin in Aysén is one end member of the oblique subduction systems on Earth with significant partitioning from the subduction zone to the surface faulting along the LOFZ and is thus similar in nature and in regards to rates to the Philippines fault (20 to 25 mm/year^16^) and in stark contrast with the San-in shear zone in Japan in the upper plate of the Nankai subduction zone where no surface faults are known^25^?

The main trace of the LOFZ (i.e. master fault) exerts a major control on volcano distribution in the SVZ (Figs. 1, 2). We discovered the Holocene active VMG and Late-Quaternary caldera (previously mapped at 1:1,000,000 as Patagonian Batholith^27^; Figs. 2, 3, 4, 5) which lies along the LOFZ. Finding the VMG and caldera was surprising, however with high rainfall and corresponding rainforest cover in addition to cloud cover, and two major icecaps still present in the area (e.g. Figs. 1, 2) it is likely that other volcanoes likely remain un-detected both in Patagonia and in similar rainforest environments globally (e.g. Alaska, Indonesia). Nevertheless, stratovolcanoes were known to be related to the LOFZ due to oblique subduction^29^ and this model is well-accepted within the community, where although the classic subducted slab model within the subduction system is still at play between the Nazca and South American Plates, the LOFZ appears to have the ultimate control on volcano distribution at the surface in the upper crust. However, it was previously believed that volcanoes occur where northeast-striking faults intersect with the main, approximately north trending fault trace of the LOFZ^29^. Our new mapping from 41.5° to 47.5° S shows that all volcanoes (irrespective of size) are found along or west of the main (or master) LOFZ (Figs. 1, 2, 3, 4), except for south of VMG, where Volcan Hudson is found. Perhaps subducted fracture or fault zones (e.g. Fig. 1) in the Nazca Plate^33^ (Fig. 1), when they meet the main trace of LOFZ in the upper plate, partially also control the location of major volcanoes. Thus both Hudson and VMG are likely within and near the northern limit of the proposed slab window (e.g.^2,3^) and/or are located above where fault/fracture zones^33^ in the subducted Nazca Plate intersect with the crustal LOFZ. Having two Late-Quaternary major calderas < 35 km apart, Hudson (~ 10 km-wide) and VMG (~ 5 km-wide), also lends strong evidence to the immense heat flow that would be expected from subduction of the CTJ, and ultimately, from the mantle upwelling that the slab window allows. Because recent thermochronological modeling suggests no evidence for reheating or thermal uplift-driven erosion and cooling within 10 to 20 Ma of slab window formation^19^, this perhaps demonstrates the important role of faults like the LOFZ play for transferring heat and fluids from the slab window to the upper crust and Earth's surface? The main trace of the LOFZ cuts the youngest, Holocene cone of the VMG and the VMG caldera, supporting an interplay between active crustal faulting and volcanism. The fast slipping main trace of the LOFZ ruptures and causes major earthquakes (at least Mw 6.2 up to at least Mw 7.7; e.g.^25,26^) and these surface ruptures cut all crustal rock units and Quaternary materials including bisecting volcanoes located along the LOFZ. The high-intensity and shallow upper crustal strong ground motions (i.e. earthquakes) generated when the LOFZ ruptures contributes to major landsliding and volcanic edifice collapse events (as found along VMG, Fig. 3). Since the LOFZ is fast slipping, this too perhaps aids in rapid transfer of heat and material from depth, because fast slipping faults tend to rupture more frequently, ruptures along the LOFZ that would lower the stress around the fault zone and perhaps allow crustal scale changes in permeability (e.g.^36,37^) at and above the brittle-ductile transition could in turn lead to eruptions along SVZ on and near the LOFZ. We are not suggesting that fault ruptures are the only mechanism for eruptions along the SVZ, only that rupture along the LOFZ may aid in the eruption of major stratovolcanoes and minor monogenetic cones within the SVZ. This may then support the idea that fault rupture along the LOFZ aids collapse of volcanoes along the LOFZ, but reduction of stress within the crust around the fault post-rupture may create conditions suitable for magma transportation to the surface along the LOFZ and eruptions that then aid in rebuilding these same volcanoes.

In addition to the crustal seismic and volcanic hazard implications, our results support the idea that the dextral LOFZ should be regarded as a major player in accommodating neotectonic deformation and magmatism in the Patagonian Andes. The LOFZ accommodates northward motion of the CM, controls distribution of volcanoes at the surface (Figs. 1, 2, 3, 4), is slipping so rapidly it is in the process of beheading LGM glacial valleys at the HC (Figs. 2, 5, 6), and likely plays a key role in the rise and fall of major stratovolcanoes (Figs. 2, 3), and is at minimum a microplate boundary fault. Our study supports the existence and helps define the location of the northern edge of the proposed slab window near VMG (which is composed of a unique unknown member of magmas with different geochemical signatures than nearby major volcanic centres based on geochemical relationships shown in Figs. 3, 4) due to subduction of the Chile Rise (i.e. CTJ) in Patagonia^2,3,7^. Along the southernmost LOFZ, the master fault appears to accommodate most of the deformation here, as revealed by the clear tectonic geomorphology, with limited slip partitioning along minor faults within the upper crust. Because this contrasts with results further north, we speculate that over the length of this long fault (~ 1200 km), the master LOFZ within the Liquiñe-Ofqui Fault System (LOFS), perhaps changes behavior from south (i.e. in our study area) to north where in the south deformation is more localised along the master fault (although still with minor subparallel structures, i.e.^23,26^) versus being more partitioned along several structures north of our study area (e.g.^17,29,38^). This is would be thus similar to the upper crustal Plate Boundary conditions in southernmost Patagonia along the Magallanes Fault where deformation is more localised along several, subparallel structures in the south that splay apart further north^35^. Clearly future high resolution remote sensing surveys focused along the master fault outlined and described here (i.e. drone/helicopter/plane structure from motion or lidar-derived topography) tied with higher resolution bedrock mapping, geochronology, and site specific Late-Quaternary glacial retreat timing based on surface exposure dating (where vegetation permits or other Quaternary dating) will help reduce uncertainties based on our new first-order strike slip geological slip-rates. However as with all science, the whole is greater than the sum of its parts, and having important new discoveries and first order rates will provide an important foundation for further work to aid our understanding of this remote natural laboratory in Patagonia. Ultimately the contemporaneous interplay between the oblique subduction of the Chile Triple Junction and formation (and migration) of a slab window and fast slipping crustal-scale strike slip faulting and volcanism in Chilean Patagonia, provides important modern insight and context to understanding paleo slab window environments in addition to plate boundary formation globally.

## Supplementary Information

Supplementary Information 1.

Supplementary Information 2.

Supplementary Information 3.

Supplementary Information 4.

## Data Availability

All data is available in the manuscript or the Supplementary Materials 1.
