# Author Correction: Liquiñe-Ofqui’s fast slipping intra-volcanic arc crustal faulting above the subducted Chile Ridge

**DOI:** 10.1038/s41598-021-03919-z

**Published:** 2021-12-17

**Authors:** Gregory P. De Pascale, Melanie Froude, Ivanna Penna, Reginald L. Hermanns, Sergio A. Sepúlveda, Daniel Moncada, Mario Persico, Gabriel Easton, Angelo Villalobos, Francisco Gutiérrez

**Affiliations:** 1 Departmento de Geología, Facultad de Ciencias Físicas y Matemáticas (FCFM), Universidad de Chile, Plaza Ercilla 803, Santiago, Chile; 2 Department of Geography, University of Sheffield, Sheffield, S10 2TN UK; 3 Geological Survey of Norway (NGU), Postboks 6315 Sluppen, 7491 Trondheim, Norway; 4 Institute of Geoscience and Petroleum, Norwegian University of Science and Technology, Trondheim, Norway; 5 Instituto de Ciencias de la Ingeniería, Universidad de O’Higgins, Libertador Bernardo O’Higgins 611, Rancagua, Chile; 6 Department of Earth Sciences, Simon Fraser University, 8888 University Drive, Burnaby, BC V5A 1S6 Canada; 7 Golder Associates, Piso 3, Las Condes, Magdalena 181, Santiago, Chile; 8 GeoExpedition, Las Barrancas 25, Pirque, Santiago, Chile

Correction to: *Scientific Reports* 10.1038/s41598-021-86413-w, published online 29 March 2021

The original version of this Article contained an error in the Referencing and with a coordinate of a field site.

Reference 21 was omitted and is listed below:

21. Melnick, D., Bookhagen, B., Strecker, M., & Echtler, H. Segmentation of megathrust rupture zones from fore-arc deformation patterns over hundreds to millions of years, Arauco peninsula, Chile. *J. Geophys. Res. Solid Earth* **114**, B01407 https://doi.org/10.1029/2008JB005788 (2009).

Consequently, the legend of Figure 1 was incomplete.

“Oblique subduction here is the driving force for dextral motion (i.e. northwards migration of the Chiloe Microplate) along the LOFZ.”

now reads:

“Oblique subduction here is the driving force for dextral motion (i.e. northwards migration of the Chiloe Microplate, after Forsythe and Nelson^10^ and Melnick et al.^21^) along the LOFZ”

As a result of the changes, the References have been renumbered accordingly.

Additionally, the lower left panel of Figure 3 contained an error in the coordinates given for the Volcan Mate Grande study site, where “45° 36′ 25″ S, 73° 05′ 40″ W” was incorrectly given as “46° 36′ 25″ S, 73° 05′ 40″ W

The original Figure 3 and accompanying legend appears below.

**Figure 3:**
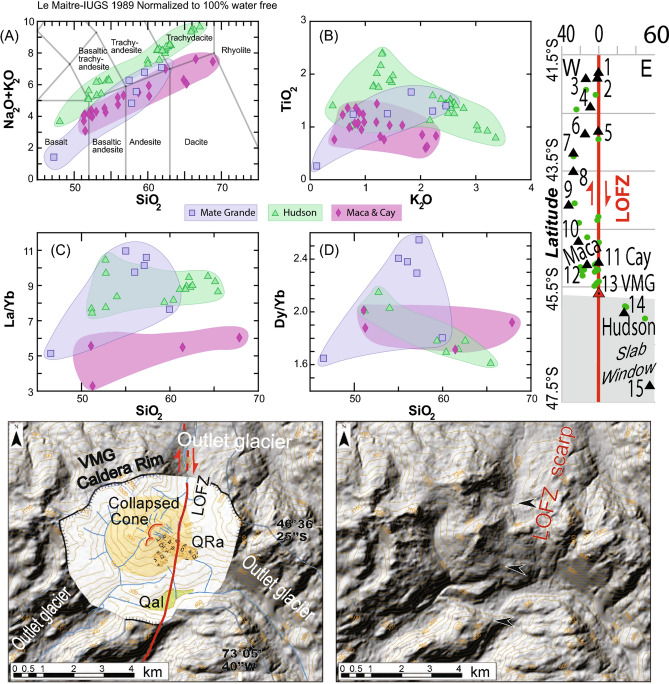
(**A**) Volcan Mate Grande (VMG: discovered during this study; 45° 35′ 28″ S, 73° 07′ 51″ W) has Hi-K calcalckaline magmas and (**B**–**D**) distinct geochemical characteristics compared with Volcan Maca and Cay complexes (*32, 33*; VMCC: typical SVZ magmas) and Volcan Hudson (VH; atypical SVZ magmas). VMG has distinctive La/Yb (**C**) and Dy/Yb (**D**) values, i.e. a completely different signature between VMG magmas and VH magmas. (**E**) Distribution (perpendicular distance (km) and normalised along-strike of the LOFZ) of stratovolcanoes and monogenetic cones from 41.5° to 47.5° S based on our mapping. (**F**,**G**) DEMs and mapping from the VMG, showing the 5 km by 4 km caldera, the young partially collapsed cone, the rock avalanche deposits (QRa), Quaternary alluvium (Qal), and the location of the main trace of the LOFZ that cuts this cone and displaces the rock avalanche deposit (likely triggered by a LOFZ earthquake/rupture) northward (i.e. dextrally) by ~ 170 ± 20 m. Base hillshade was generated with Esri ArcMap v.10.3 software (under fair terms of use, https://www.esri.com/en-us/legal/copyright-trademarks)^38^ using a digital elevation model downloaded from ALOS PALSAR Global Radar Imagery^39^ with 12.5 m resolution (https://asf.alaska.edu/data-sets/sar-data-sets/alos-palsar/).

The original Article has been corrected.

