# Supplementary material for: Liquiñe-Ofqui’s fast slipping intra-volcanic arc crustal faulting above the subducted Chile Ridge
Source: Sci Rep. 2021 Mar 29;11:7069. doi: 10.1038/s41598-021-86413-w (PMC8007613; doi:10.1038/s41598-021-86413-w)
Supplement: Supplementary file 2 — Supplementary Information 2. [file 41598_2021_86413_MOESM2_ESM.pdf]

ALS Patagonia S.A.

Hermanos Carrera Pinto 159,
Parque Industrial Los Libertadores, Colina
Santiago
Phone: +56 (2) 26546100 Fax: +56 (2) 26546163
www.alsglobal.com/geochemistry

To: GREGORY DE PASCALE
PLAZA ERCILLA 803
SANTIAGO,SANTIAGO RM

Finalized Date: 3-DEC-2018
This copy reported on 5-DEC-2018
Account: PASCALE

REPORT LS18286137

This report is for 7 Rock samples submitted to our lab in La Serena, IV, Chile on 16-NOV-2018.

The following have access to data associated with this report:

GREG DE PASCALE		

SAMPLE PREPARATION

ALS CODE	DESCRIPTION
WEI-21	Received Sample Weight
LOG-22	Sample login - Rcd w/o BarCode
BAG-01	Bulk Master for Storage
CRU-QC	Crushing QC Test
PUL-QC	Pulverizing QC Test
CRU-31	Fine crushing - 70% <2mm
SPL-21	Split sample - riffle splitter
PUL-32	Pulverize 1000g to 85% < 75 um

ANALYTICAL PROCEDURES

ALS CODE	DESCRIPTION	
ME-MS61r	4A multi-element ICP-MS + REE	
ME-XRF26	Whole Rock By Fusion/XRF	XRF
OA-GRA05x	LOI for XRF	WST-SEQ

This is the Final Report and supersedes any preliminary report with this report number. Results apply to samples as submitted. All pages of this report have been checked and approved for release.

***** See Appendix Page for comments regarding this report *****

Signature:

Rene Mamani, Laboratory Manager, Peru

ALS Patagonia S.A.

Hermanos Carrera Pinto 159,
Parque Industrial Los Libertadores, Colina
Santiago
Phone: +56 (2) 26546100 Fax: +56 (2) 26546163
www.alsglobal.com/geochemistry

To: GREGORY DE PASCALE
PLAZA ERCILLA 803
SANTIAGO,SANTIAGO RM

Finalized Date: 3-DEC-2018
Account: PASCALE

TEST REPORT LS18286137

Sample Description	Method	WEI-21	ME-XRF26	ME-XRF26	ME-XRF26	ME-XRF26	ME-XRF26	ME-XRF26	ME-XRF26	ME-XRF26	ME-XRF26	ME-XRF26	ME-XRF26	ME-XRF26	ME-XRF26	ME-XRF26
	Analyte	Recvd Wt.	Al2O3	BaO	CaO	Cr2O3	Fe2O3	K2O	MgO	MnO	Na2O	P2O5	SO3	SiO2	SrO	TiO2
	Units	kg	%	%	%	%	%	%	%	%	%	%	%	%	%	%
	LOD	0.02	0.01	0.01	0.01	0.01	0.01	0.01	0.01	0.01	0.01	0.01	0.01	0.01	0.01	0.01
QF1		0.20	16.20	0.08	5.19	<0.01	7.21	2.22	2.61	0.16	4.31	0.46	0.34	57.33	0.06	1.30
QF2		0.56	15.87	0.07	5.39	<0.01	9.10	1.83	2.95	0.15	4.18	0.51	0.01	55.01	0.06	1.66
QF3		0.84	15.85	0.07	3.78	<0.01	7.18	2.46	1.92	0.17	4.41	0.51	<0.01	59.95	0.06	1.40
QF4		0.39	17.04	0.07	6.49	<0.01	7.94	0.78	3.77	0.15	3.90	0.37	0.14	56.02	0.07	1.24
QF5		0.28	19.90	0.02	10.60	<0.01	10.26	0.12	10.35	0.16	1.28	0.04	0.10	46.52	0.06	0.26
QF6		0.79	16.78	0.09	6.41	<0.01	7.63	1.40	3.32	0.14	4.03	0.41	0.47	57.09	0.07	1.25
LSR-1		0.21	13.78	0.05	1.83	<0.01	5.44	1.16	1.76	0.12	2.68	0.14	<0.01	70.08	0.03	0.57

***** See Appendix Page for comments regarding this report *****

ALS Patagonia S.A.

Hermanos Carrera Pinto 159,
Parque Industrial Los Libertadores, Colina
Santiago
Phone: +56 (2) 26546100 Fax: +56 (2) 26546163
www.alsglobal.com/geochemistry

To: GREGORY DE PASCALE
PLAZA ERCILLA 803
SANTIAGO,SANTIAGO RM

Finalized Date: 3-DEC-2018
Account: PASCALE

TEST REPORT LS18286137

Sample Description	ME-XRF26	OA-GRA05x	ME-MS61r	ME-MS61r	ME-MS61r	ME-MS61r	ME-MS61r	ME-MS61r	ME-MS61r	ME-MS61r	ME-MS61r	ME-MS61r	ME-MS61r	ME-MS61r	ME-MS61r
Analyte	Total	LOI 1000	Ag	Al	As	Ba	Be	Bi	Ca	Cd	Ce	Co	Cr	Cs	Cu
Units	%	%	ppm	%	ppm	ppm	ppm	ppm	%	ppm	ppm	ppm	ppm	ppm	ppm
LOD	0.01	0.01	0.01	0.01	0.2	10	0.05	0.01	0.01	0.02	0.01	0.1	1	0.05	0.2
QF1	99.33	1.77	0.06	8.07	1.9	590	1.95	0.04	3.66	0.07	51.7	12.4	13	1.00	21.9
QF2	98.69	1.80	0.03	8.01	1.5	490	1.79	0.06	3.72	0.08	59.7	19.6	3	0.59	34.9
QF3	99.86	2.01	0.06	8.04	1.4	550	2.09	0.08	2.68	0.13	69.2	10.4	<1	0.76	15.2
QF4	100.45	2.39	0.06	8.35	2.2	430	1.26	0.15	4.41	0.09	43.7	19.6	27	0.60	44.7
QF5	101.25	1.48	0.07	9.51	1.2	40	0.21	0.04	7.16	0.08	4.08	52.2	6	0.13	81.6
QF6	101.45	2.28	0.03	8.28	1.4	680	1.83	0.06	4.42	0.05	46.5	15.5	26	0.84	26.3
LSR-1	100.15	2.46	0.06	7.15	3.6	340	1.10	0.09	1.34	0.06	36.6	12.1	19	4.05	30.2

***** See Appendix Page for comments regarding this report *****

ALS Patagonia S.A.

Hermanos Carrera Pinto 159,
Parque Industrial Los Libertadores, Colina
Santiago
Phone: +56 (2) 26546100 Fax: +56 (2) 26546163
www.alsglobal.com/geochemistry

To: **GREGORY DE PASCALE**
PLAZA ERCILLA 803
SANTIAGO,SANTIAGO RM

Finalized Date: 3-DEC-2018
Account: PASCALE

TEST REPORT LS18286137

Sample Description	Method	ME-MS61r	ME-MS61r	ME-MS61r	ME-MS61r	ME-MS61r	ME-MS61r	ME-MS61r	ME-MS61r	ME-MS61r	ME-MS61r	ME-MS61r	ME-MS61r	ME-MS61r	ME-MS61r	ME-MS61r
	Analyte	Fe	Ga	Ge	Hf	In	K	La	Li	Mg	Mn	Mo	Na	Nb	Ni	P
	Units	%	ppm	ppm	ppm	ppm	%	ppm	ppm	%	ppm	ppm	%	ppm	ppm	ppm
	LOD	0.01	0.05	0.05	0.1	0.005	0.01	0.5	0.2	0.01	5	0.05	0.01	0.1	0.2	10
QF1		4.93	21.0	0.12	2.1	0.083	1.83	23.1	10.7	1.48	1160	0.71	3.21	11.7	7.2	2060
QF2		6.12	20.9	0.17	3.7	0.078	1.49	27.3	13.4	1.69	1080	0.76	3.16	13.5	5.5	2270
QF3		4.86	21.1	0.14	7.4	0.091	2.00	31.9	31.4	1.08	1240	0.49	3.21	14.6	0.5	2280
QF4		5.25	21.5	0.12	3.3	0.072	0.62	20.3	21.0	2.10	1080	0.74	2.78	8.8	20.4	1570
QF5		7.06	15.25	0.12	0.2	0.064	0.08	1.9	7.8	6.03	1120	0.13	0.92	0.5	93.5	130
QF6		5.13	21.2	0.12	3.5	0.078	1.13	22.1	12.3	1.87	1010	0.74	2.91	10.1	18.8	1780
LSR-1		3.72	16.25	0.10	2.2	0.039	0.96	20.2	22.9	1.03	854	0.19	1.97	5.2	10.8	630

***** See Appendix Page for comments regarding this report *****

ALS Patagonia S.A.

Hermanos Carrera Pinto 159,
Parque Industrial Los Libertadores, Colina
Santiago
Phone: +56 (2) 26546100 Fax: +56 (2) 26546163
www.alsglobal.com/geochemistry

To: GREGORY DE PASCALE
PLAZA ERCILLA 803
SANTIAGO,SANTIAGO RM

Finalized Date: 3-DEC-2018
Account: PASCALE

TEST REPORT LS18286137

Sample Description	Method<br>Analyte<br>Units<br>LOD	ME-MS61r<br>Pb<br>ppm<br>0.5	ME-MS61r<br>Rb<br>ppm<br>0.1	ME-MS61r<br>Re<br>ppm<br>0.002	ME-MS61r<br>S<br>%<br>0.01	ME-MS61r<br>Sb<br>ppm<br>0.05	ME-MS61r<br>Sc<br>ppm<br>0.1	ME-MS61r<br>Se<br>ppm<br>1	ME-MS61r<br>Sn<br>ppm<br>0.2	ME-MS61r<br>Sr<br>ppm<br>0.2	ME-MS61r<br>Ta<br>ppm<br>0.05	ME-MS61r<br>Te<br>ppm<br>0.05	ME-MS61r<br>Th<br>ppm<br>0.01	ME-MS61r<br>Ti<br>%<br>0.005	ME-MS61r<br>Tl<br>ppm<br>0.02	ME-MS61r<br>U<br>ppm<br>0.1
QF1		12.7	40.3	<0.002	0.14	0.10	18.5	1	1.6	486	0.65	<0.05	2.35	0.758	0.32	0.4
QF2		10.0	37.1	<0.002	<0.01	0.08	22.6	1	1.6	492	0.71	<0.05	2.86	0.957	0.23	0.7
QF3		15.7	62.7	<0.002	<0.01	0.09	17.2	1	2.0	470	0.84	<0.05	6.44	0.813	0.33	1.8
QF4		20.0	12.0	<0.002	0.05	0.11	21.3	1	1.4	581	0.51	<0.05	2.87	0.696	0.14	0.7
QF5		3.0	1.7	<0.002	0.03	0.32	13.5	1	0.6	482	<0.05	0.09	0.18	0.142	0.02	0.1
QF6		8.7	23.5	<0.002	0.19	0.09	20.9	1	1.4	580	0.56	0.07	2.76	0.713	0.20	0.8
LSR-1		9.9	40.8	<0.002	<0.01	0.41	13.0	1	1.2	254	0.37	<0.05	6.34	0.335	0.31	1.4

***** See Appendix Page for comments regarding this report *****

ALS Patagonia S.A.

Hermanos Carrera Pinto 159,
Parque Industrial Los Libertadores, Colina
Santiago
Phone: +56 (2) 26546100 Fax: +56 (2) 26546163
www.alsglobal.com/geochemistry

To: **GREGORY DE PASCALE**
PLAZA ERCILLA 803
SANTIAGO,SANTIAGO RM

Finalized Date: 3-DEC-2018
Account: PASCALE

TEST REPORT LS18286137

Sample Description	Method<br>Analyte<br>Units<br>LOD	ME-MS61r<br>V<br>ppm<br>1	ME-MS61r<br>W<br>ppm<br>0.1	ME-MS61r<br>Y<br>ppm<br>0.1	ME-MS61r<br>Zn<br>ppm<br>2	ME-MS61r<br>Zr<br>ppm<br>0.5	ME-MS61r<br>Dy<br>ppm<br>0.05	ME-MS61r<br>Er<br>ppm<br>0.03	ME-MS61r<br>Eu<br>ppm<br>0.03	ME-MS61r<br>Gd<br>ppm<br>0.05	ME-MS61r<br>Ho<br>ppm<br>0.01	ME-MS61r<br>Lu<br>ppm<br>0.01	ME-MS61r<br>Nd<br>ppm<br>0.1	ME-MS61r<br>Pr<br>ppm<br>0.03	ME-MS61r<br>Sm<br>ppm<br>0.03	ME-MS61r<br>Tb<br>ppm<br>0.01
QF1		142	0.4	26.5	88	74.0	5.55	2.62	1.80	6.23	1.03	0.31	29.4	6.85	6.14	0.92
QF2		218	0.3	28.1	91	118.5	5.99	2.86	2.06	6.95	1.12	0.33	34.3	8.01	7.01	0.98
QF3		105	0.4	38.3	95	277	7.52	4.20	2.14	8.29	1.53	0.64	37.1	9.19	7.78	1.18
QF4		158	0.4	22.3	100	99.8	4.95	2.43	1.62	5.82	0.91	0.30	25.5	5.70	5.37	0.81
QF5		125	<0.1	3.1	87	6.2	0.61	0.35	0.46	0.56	0.12	0.06	2.4	0.57	0.53	0.09
QF6		149	0.3	25.5	66	109.5	5.00	2.52	1.69	5.83	0.97	0.30	26.6	6.09	5.59	0.80
LSR-1		73	0.7	17.2	65	80.9	3.04	1.76	0.96	3.28	0.62	0.29	16.5	4.23	3.25	0.46

***** See Appendix Page for comments regarding this report *****

ALS Patagonia S.A.

Hermanos Carrera Pinto 159,
Parque Industrial Los Libertadores, Colina
Santiago
Phone: +56 (2) 26546100 Fax: +56 (2) 26546163
www.alsglobal.com/geochemistry

To: **GREGORY DE PASCALE**
PLAZA ERCILLA 803
SANTIAGO,SANTIAGO RM

Finalized Date: 3-DEC-2018
Account: PASCALE

TEST REPORT LS18286137

Sample Description	Method<br>Analyte<br>Units<br>LOD	ME-MS61r<br>Tm<br>ppm<br>0.01	ME-MS61r<br>Yb<br>ppm<br>0.03
QF1		0.36	2.18
QF2		0.41	2.49
QF3		0.64	4.17
QF4		0.35	2.08
QF5		0.06	0.37
QF6		0.36	2.18
LSR-1		0.26	1.65

***** See Appendix Page for comments regarding this report *****

ALS Patagonia S.A.
Hermanos Carrera Pinto 159,
Parque Industrial Los Libertadores, Colina
Santiago
Phone: +56 (2) 26546100 Fax: +56 (2) 26546163
www.alsglobal.com/geochemistry

To: GREGORY DE PASCALE
PLAZA ERCILLA 803
SANTIAGO,SANTIAGO RM

Finalized Date: 3-DEC-2018
Account: PASCALE

TEST REPORT LS18286137

REPORT COMMENTS

ANALYTICAL COMMENTS

REE's may not be totally soluble in this method.

Applies to Method: ME-MS61r

LABORATORY ADDRESSES

Processed at ALS Lima located at Calle 1 LT-1A Mz-D, esq. Calle A, Urb. Industrial Bocanegra Callao 01, Lima, Peru.

Applies to Method: ME-MS61r, ME-XRF26, OA-GRA05x

Processed at ALS La Serena located at Avenida La Fragua 1130, Barrio Industrial Chanar, Coquimbo, IV, Chile.

Applies to Method:

BAG-01	CRU-31	CRU-QC	LOG-22
PUL-32	PUL-QC	SPL-21	WEI-21
