# Supplementary material for: Liquiñe-Ofqui’s fast slipping intra-volcanic arc crustal faulting above the subducted Chile Ridge
Source: Sci Rep. 2021 Mar 29;11:7069. doi: 10.1038/s41598-021-86413-w (PMC8007613; doi:10.1038/s41598-021-86413-w)
Supplement: Supplementary file 4 — Supplementary Information 4. [file 41598_2021_86413_MOESM4_ESM.pdf]

Supplementary Material

Liquiñe-Ofqui's fast slipping intra-volcanic arc crustal faulting above the subducted Chile Ridge

Gregory P. De Pascale, Melanie Froude, Ivanna Penna, Reginald L Hermanns, Sergio A Sepúlveda, Daniel Moncada, Mario Persico, Gabriel Easton, Angelo Villalobos, Francisco Gutiérrez

Correspondence to: snowyknight@gmail.com

This PDF file includes:

Materials
Supplementary Text
Figs. S1 to S23
Tables S1 to S2
Captions for Data S1 to S2

Other Supplementary Materials for this manuscript include the following:

Data S1 – SfM model
Data S2 – Geochemistry data from MGV in Excel

Supplemental References

Additional Observations

This section presents addition data including high resolution photos (Figures S5-S14) regarding the Mate Grande Volcano (or Volcan Mate Grande) that we discovered during this investigation. Maps were made here under fair use using the software Google Earth[1]. These photos were taken by helicopter and by drone. We knew it was difficult to map the LOFZ north of the Huemules Cirque, however we were surprised to know that the reason north of the Quitralco Fiord was a previously undiscovered volcano that we call the Volcan Mate Grande (Mate Grande Volcano). In addition, Table S2 outlines some of the basic information and observations about this volcano based on our investigation. VMG samples (lava and tuff) were collected at the Quitralco Fiord.

Additional field evidence from the Huemules Cirque including field photos are shown in Figures S15-S17. Additionally field photos from along the LOFZ from the Laguna San Rafael are shown in Figures S18-S21. Examples from the structure from motion (SfM) model from the Huemules Cirque are shown in Figures S21-S22. These aerial photographs (taken from helicopter by the authors) were modelled using Agisoft Standard Photoscan Pro 1.3.2 (2018)[2] in order to develop Structure from Motion (SfM) site models including 3D DEMs (which include vegetation and thus could be referred to as Digital Surface Models (DSM)).

Please note that geochemical sample LSR-1 is from another study (as shown in the lab report) but was processed in the same batch as samples QF1-QF6.

Fig. S1.

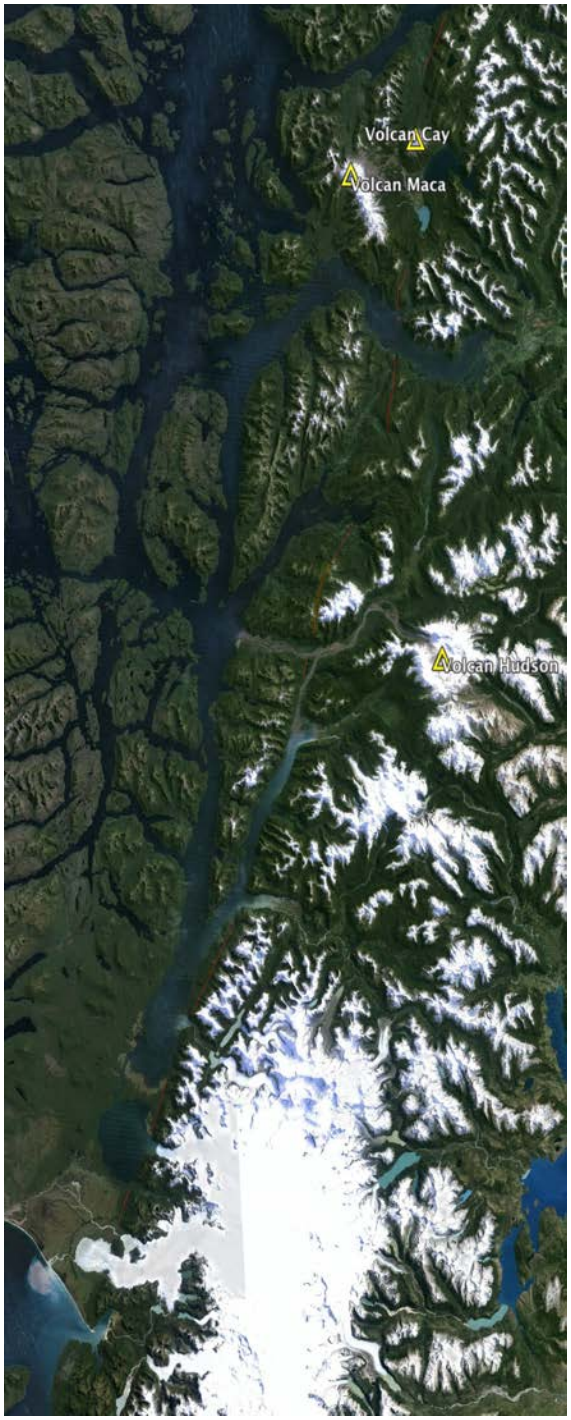

Onshore main trace of the LOFZ mapped from the Ofqui Peninsula in the South to the Puyhaipue Canal in the North shown over Google Earth Imagery. Fault traces in red and correspond with Table S1 below. Known major volcanos shown in yellow. Note lack of fault mapping NW of Hudson. This corresponds with the Volcan Mate Grande. Large white area is the Northern Patagonia Icecap. Here although much of the LOFZ is onshore, onshore traces are mostly clear. Mapping over images used under fair use from Google Earth[1].

Fig. S2.

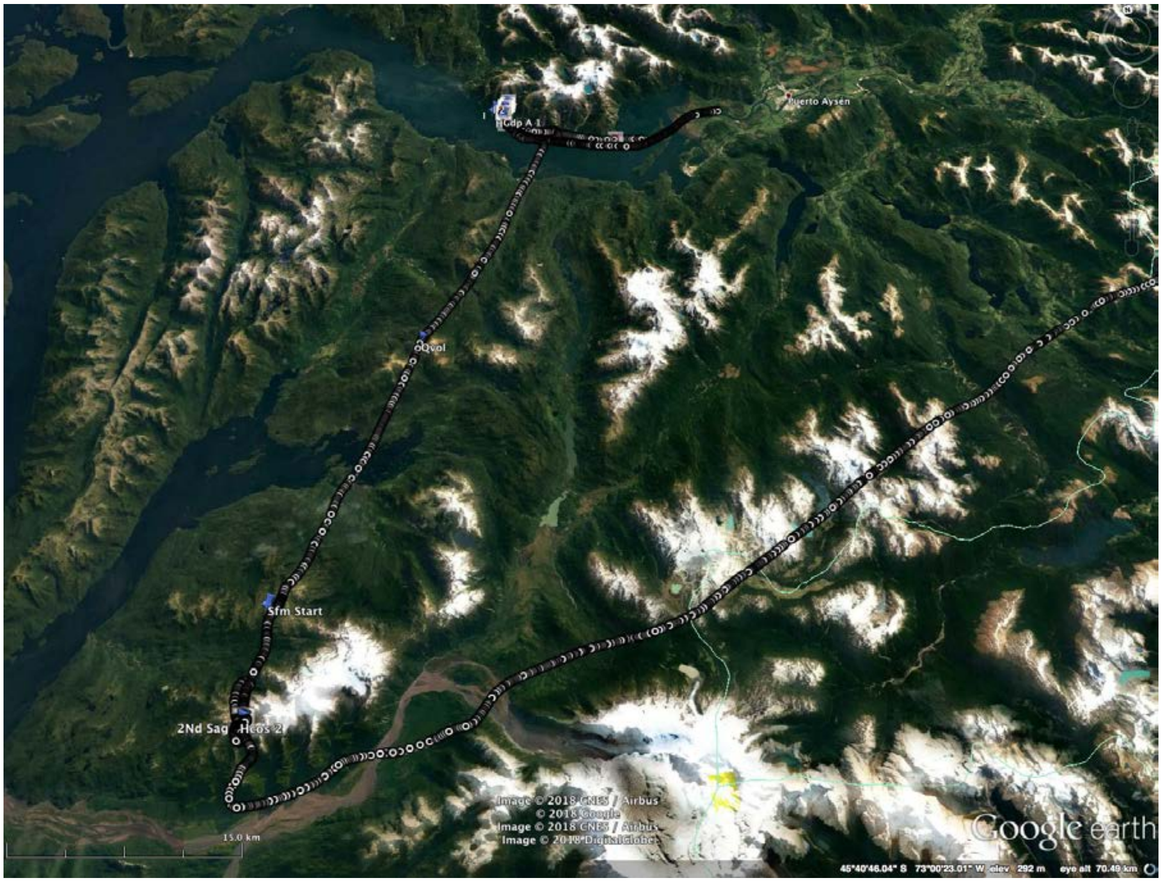

Google Earth Image showing the GPS track log of the helicopter reconnaissance. Note we flew counterclockwise along the flanks of the Hudson Volcano, went to the Huemules Cirque, then flew along the LOFZ, crossing the Mate Grande Volcano and up to Aysen Fiord. Mapping over images used under fair use from Google Earth[1] https://www.google.com/earth/.

Fig. S3.

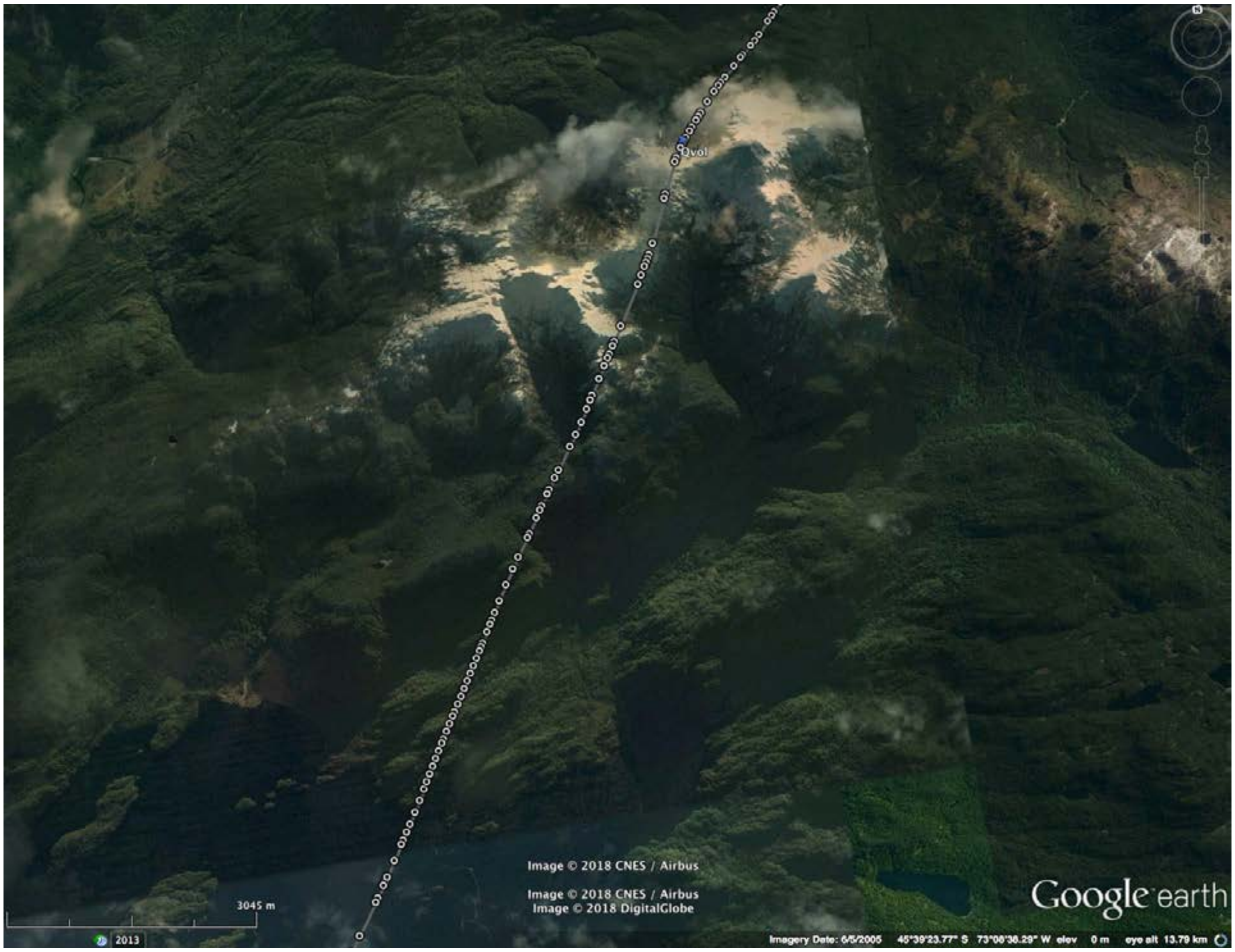

Map showing the track log (GPS) of our helicopter flight when we flew over and photographed the Mate Grande Volcano (VMG). This was our flight between the Huemules Cirque and Aysen Fiord. Qvol is a GPS point noting that we saw Quaternary Volcanics during the helicopter reconnaissance. This is at the location of Volcano Mate Grande with helicopter photographs below. Note how the snow in the Google Earth images could help conceal type of lithology here. Mapping over images used under fair use from Google Earth[1] https://www.google.com/earth/.

Fig. S4.

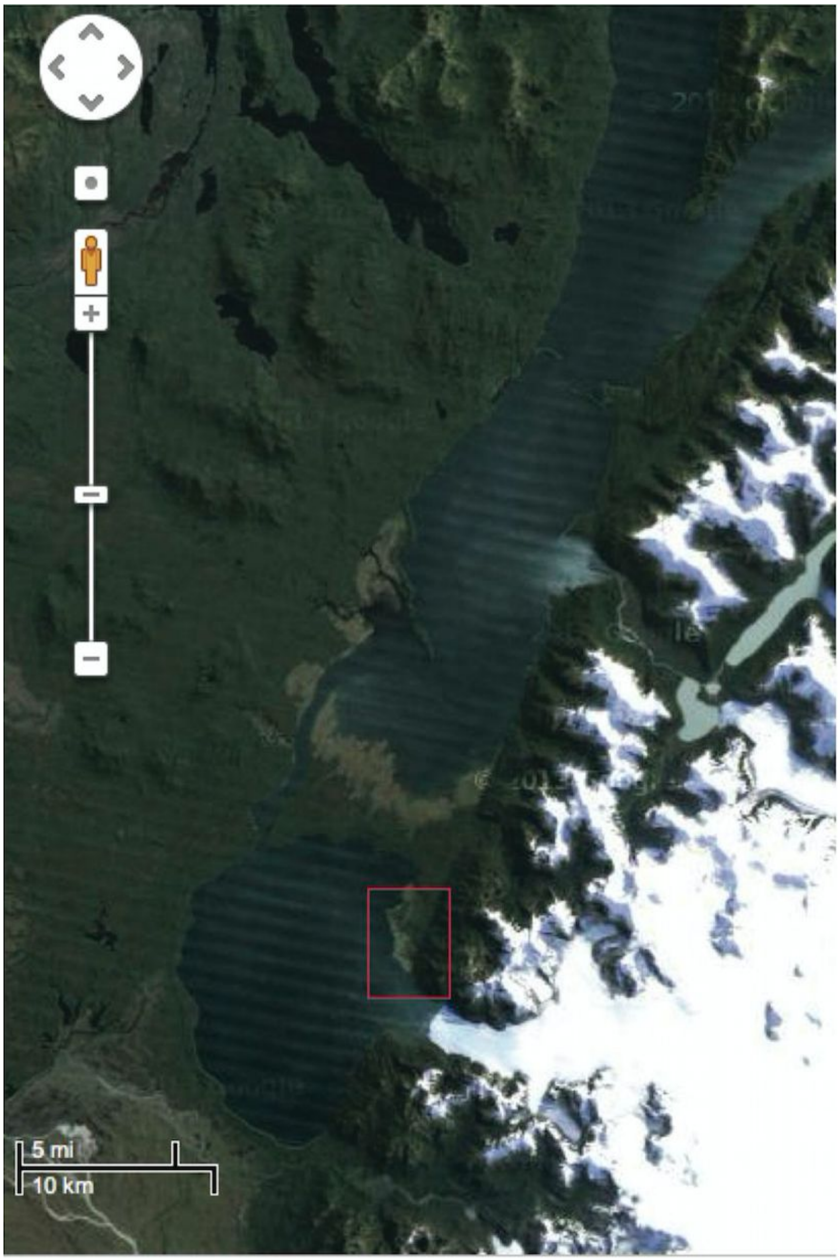

Area visited by boat in the Laguna San Rafael area shown on Google Earth Imagery. The LOFZ can be seen clearly as the rangefront for the Southern Andes here. Note the tidewater San Rafael Glacier which is a valley glacier of the Northern Patagonian Icecap. In the area shown with the red box, fault rocks and short displacements, shown in photos below were documented in the field coincident with the geomorphic trace. Mapping over images used under fair use from Google Earth[1] https://www.google.com/earth/.

Fig. S5.

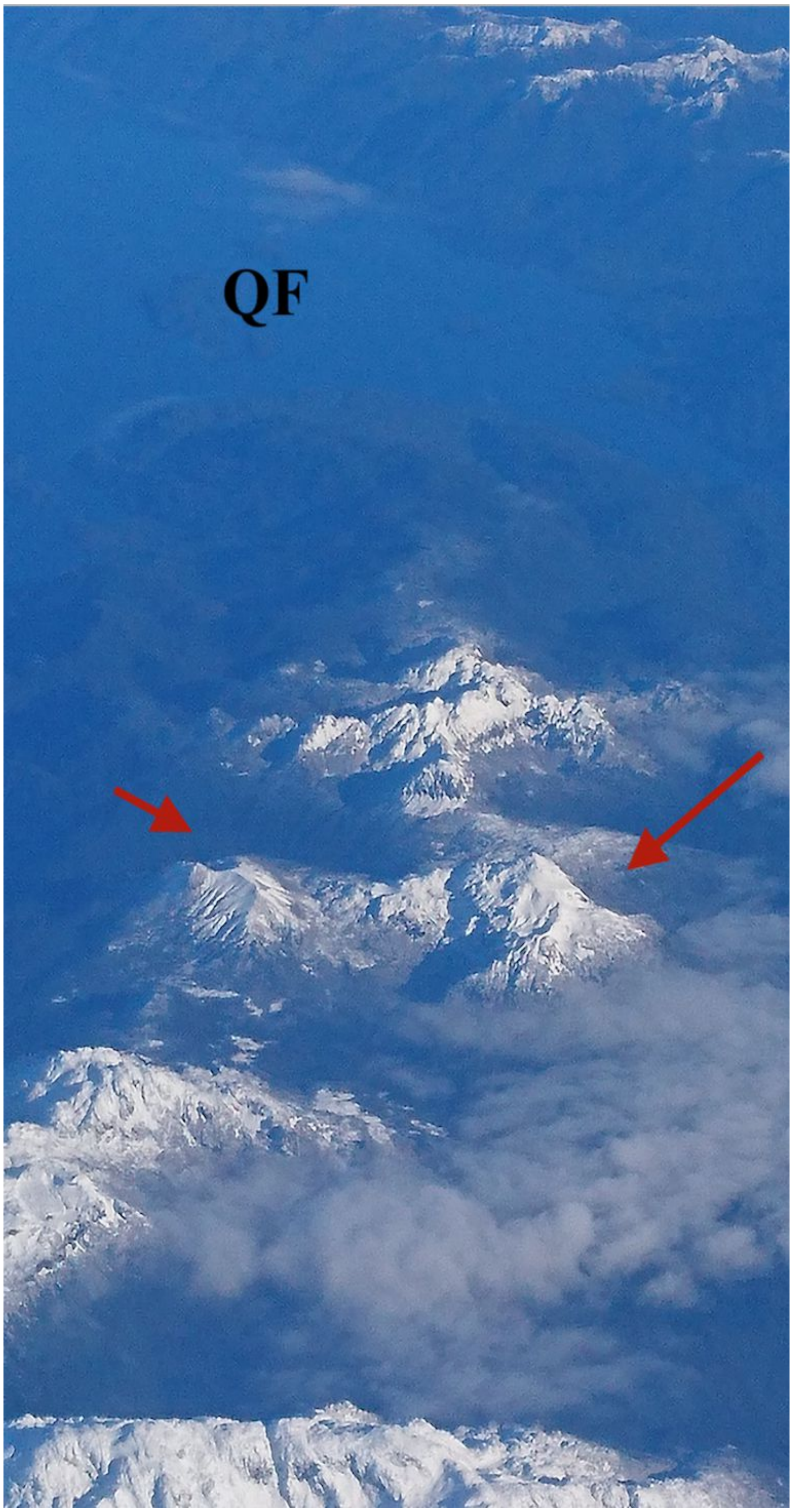

Oblique airphoto of the Volcan Mate Grande taken from a commercial aircraft window in June 2018 looking Southwest. Arrows point to the zones visible in the main paper Fig. 2. QF stands for Quitralco Fiord. Helicopter photos in the figures below show this site without snow cover. Photo by De Pascale.

Fig. S6.

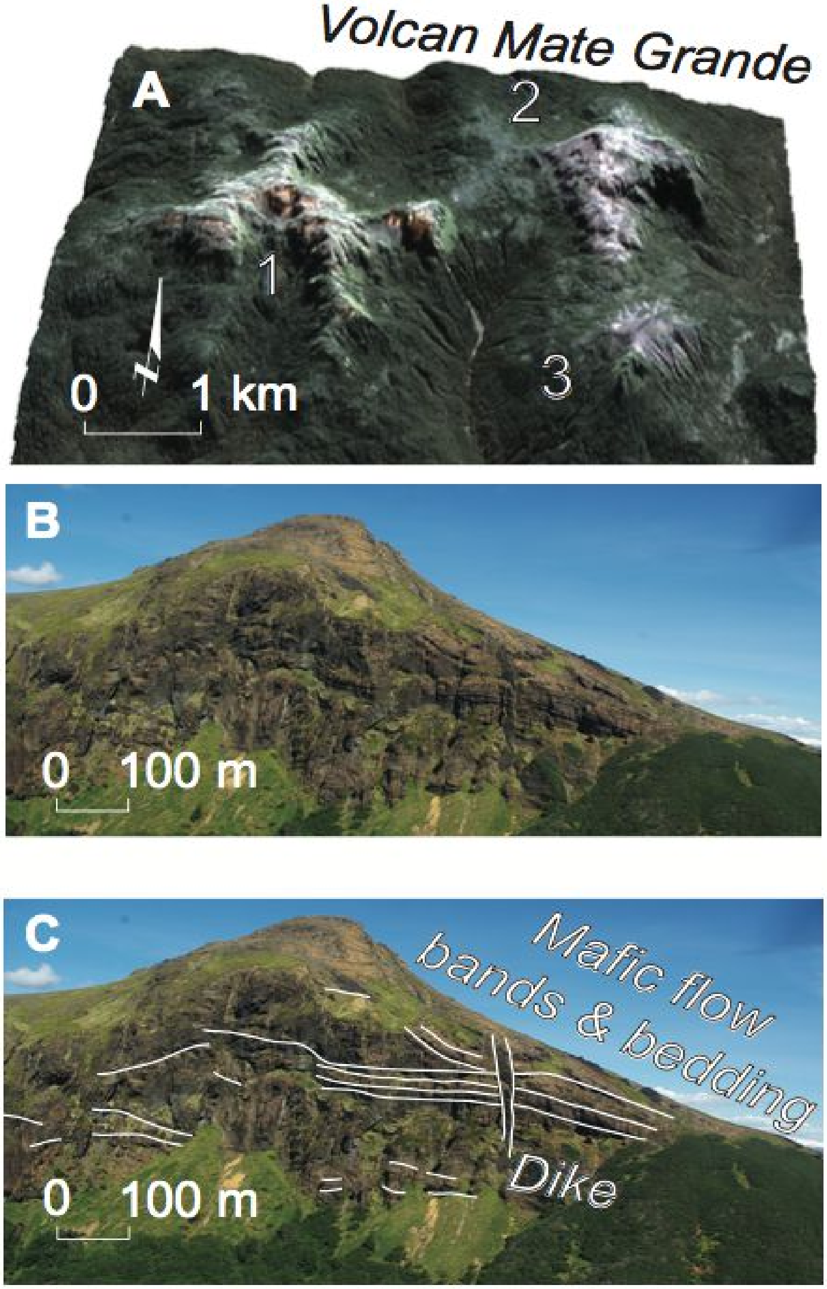

Oblique image of the Mate Grande Volcano (VMG) and annotated photos collected during helicopter reconnaissance. Note the circular caldera edge to the left of #3.

Fig. S7.

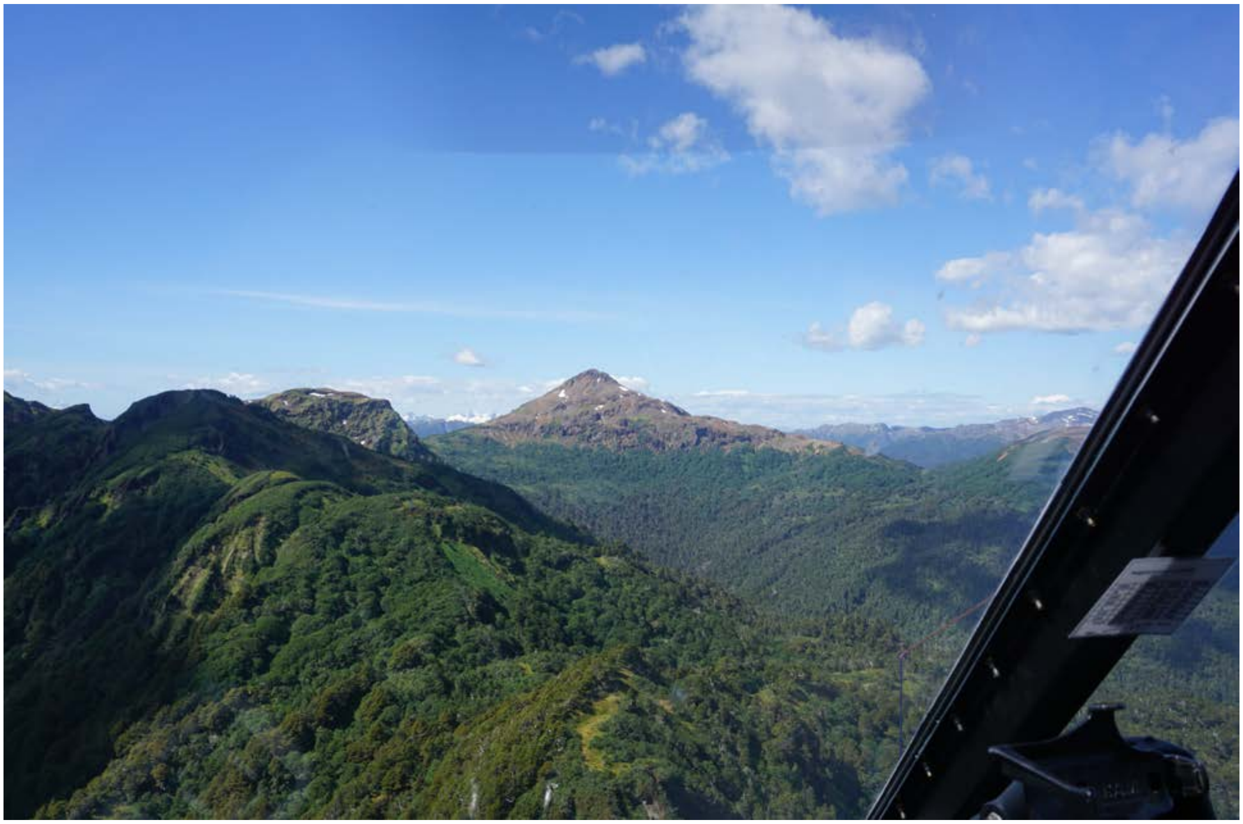

Photograph taken by helicopter while mapping the LOFZ. Flying over the volcanic ridge of the Mate Grande Volcano we were surprised as this area is mapped as “Patagonian Batholith - Mg”. Main edifice in center shown in Figure 3 of the main paper.

Fig. S8.

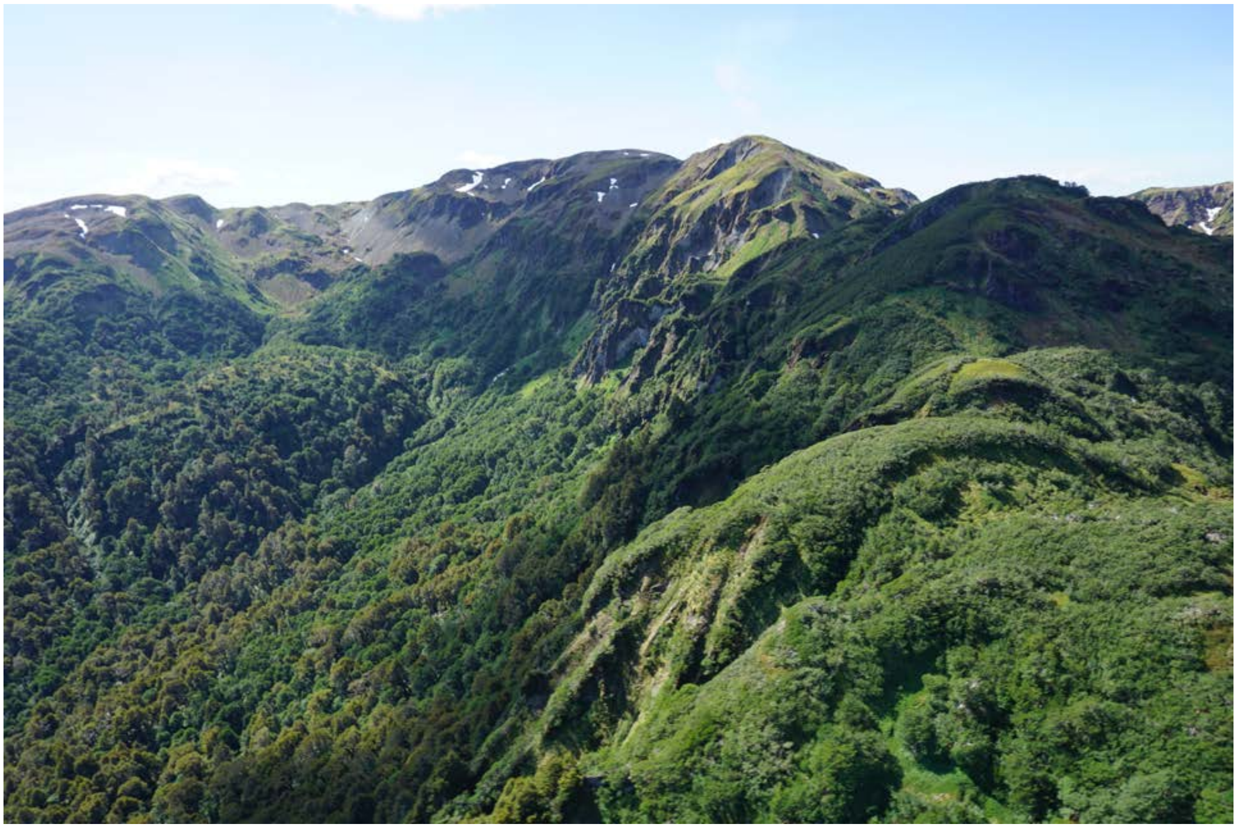

Flying over the volcanic ridge within the Mate Grande Volcano. Note the scalloped appearance from weak volcanic materials eroding here.

Fig. S9.

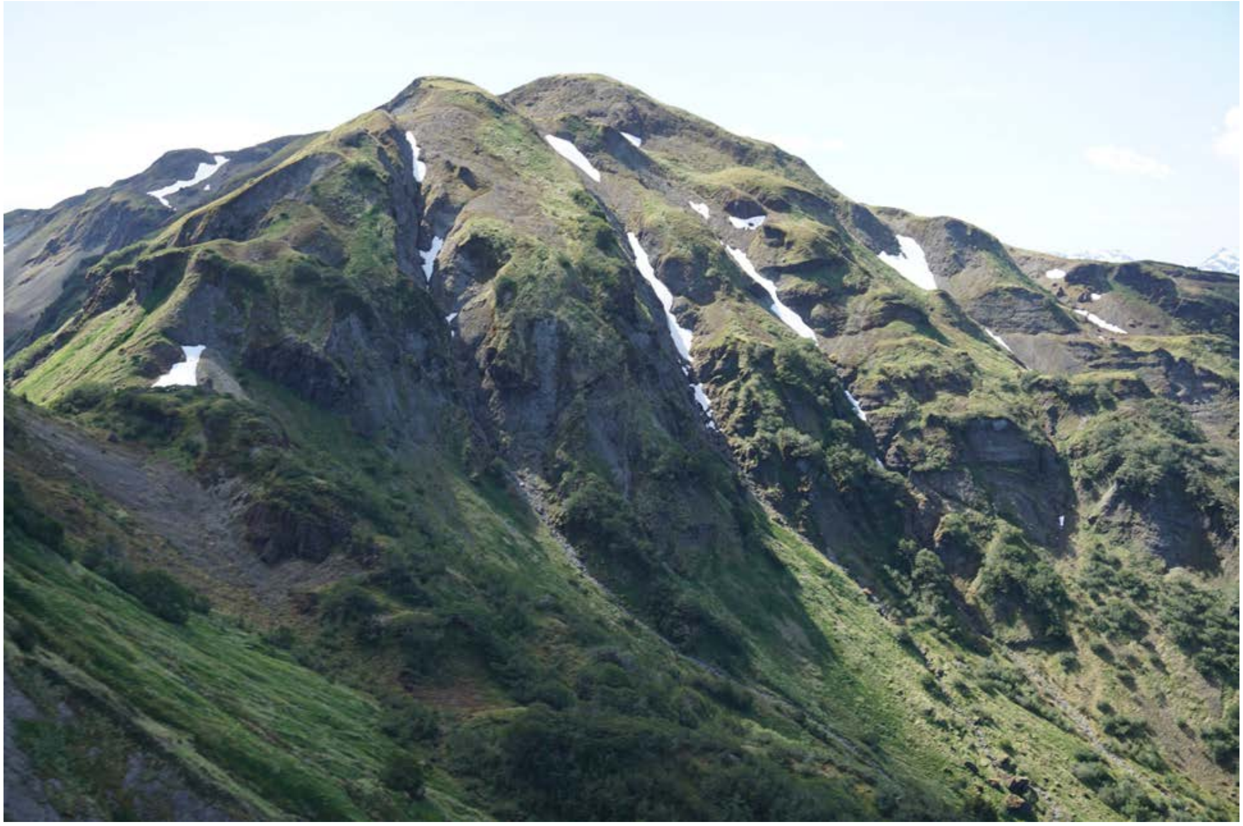

Helicopter photograph of the Mate Grande Volcano. Note the stratified, sub-horizontal beds and lava with coarse pyroclastic flow bedding. Note also the retrogressive gullying of the soft materials and lack of evidence of glaciation here. When zoomed in some layers appear blocky – likely due to pyroclastic flows.

Fig. S10.

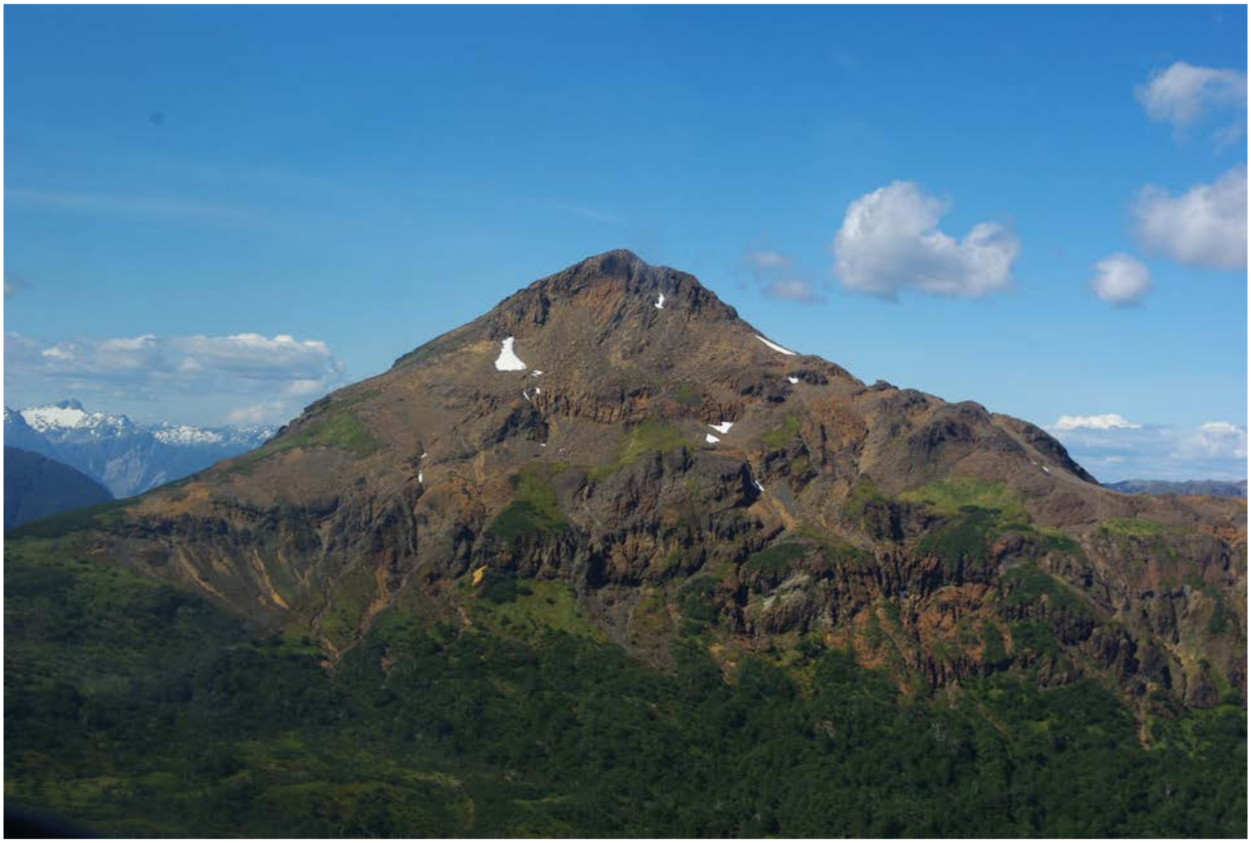

Helicopter photograph of the Mate Grande Volcano. Note the mafic (dark) colours, and yellow colours associated with hydrothermal alteration that is common in volcanic systems. Also note the bedding, sub-horizontal here which of course is not present in intrusive rocks like is found in the nearby Patagonian batholith.

Fig. S11.

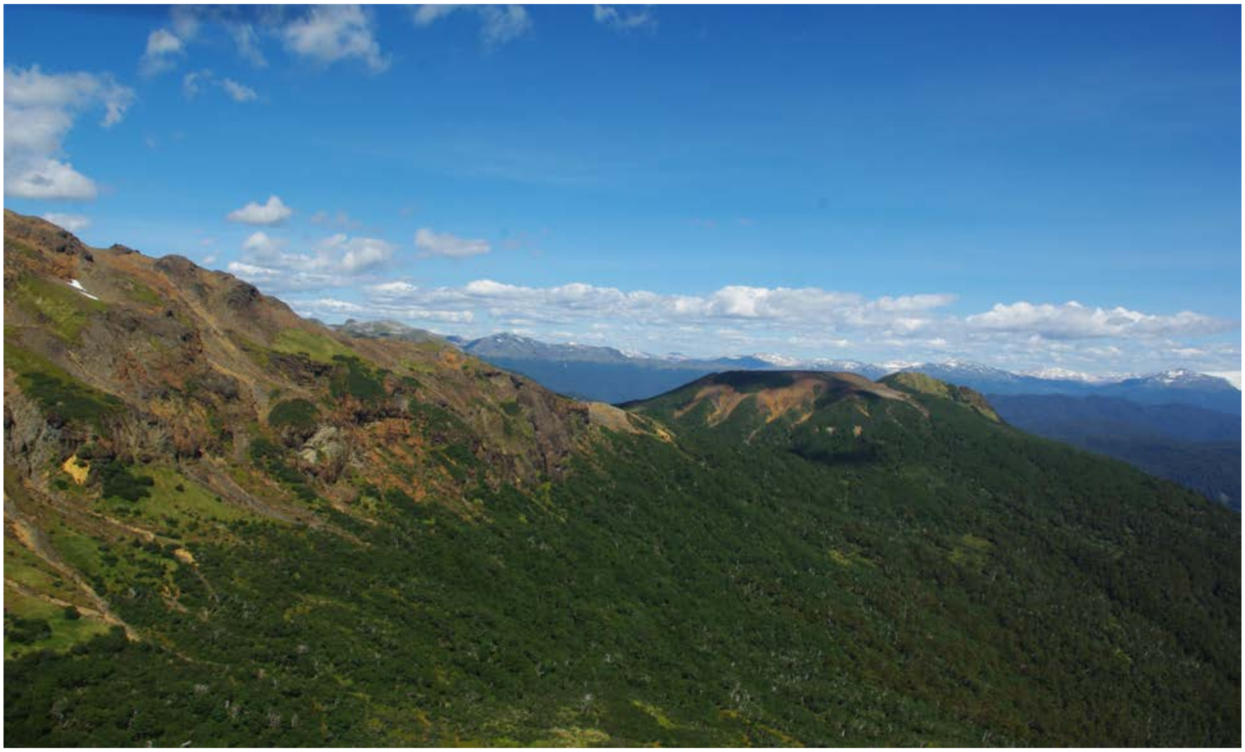

Helicopter photograph of the Mate Grande Volcano. Note the mafic (dark) colours, and bright yellow colours associated with hydrothermal alteration that is common in volcanic systems. Low hill in the background is the backside of the youngest cone shown in Figure 2 of the main paper.

Fig. S12.

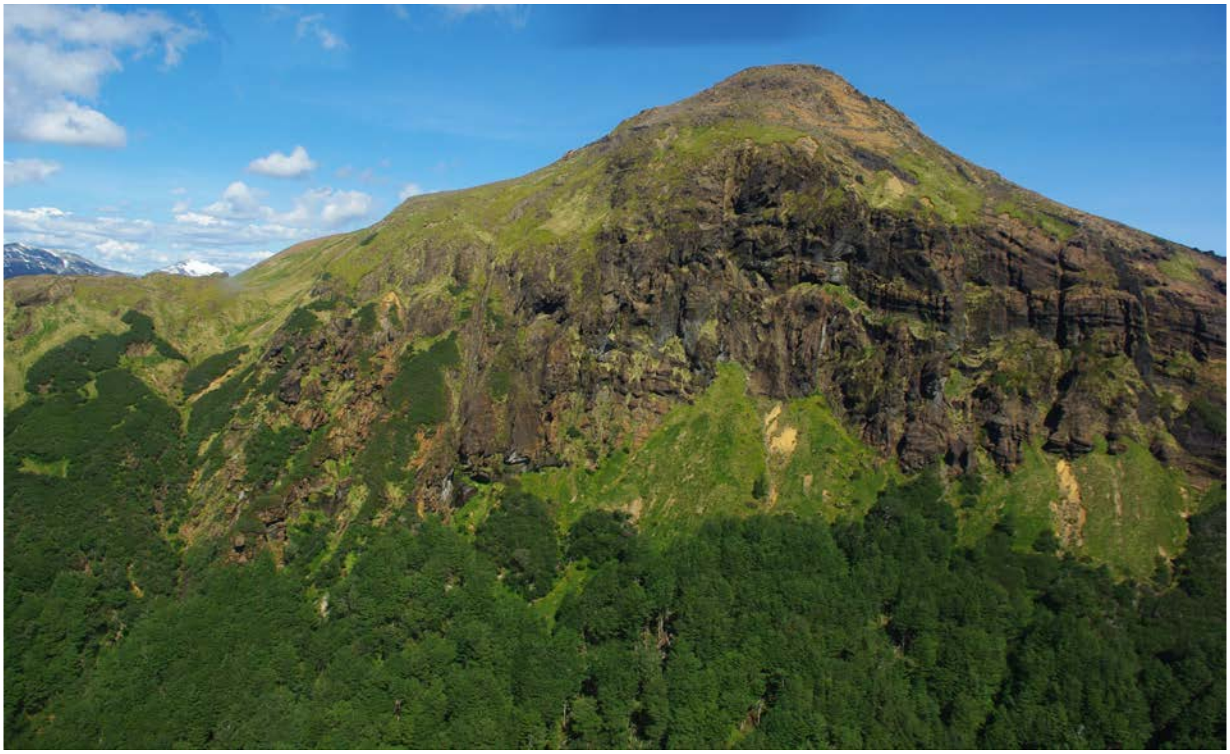

Helicopter photograph of the Mate Grande Volcano. Note the mafic (dark) colours, and yellow colours associated with hydrothermal alteration that is common in volcanic systems. Note the subhorizontal bedding and flow bands as well. This photo is slightly further north from the photo shown in the main paper (Fig. 2).

Fig. S13.

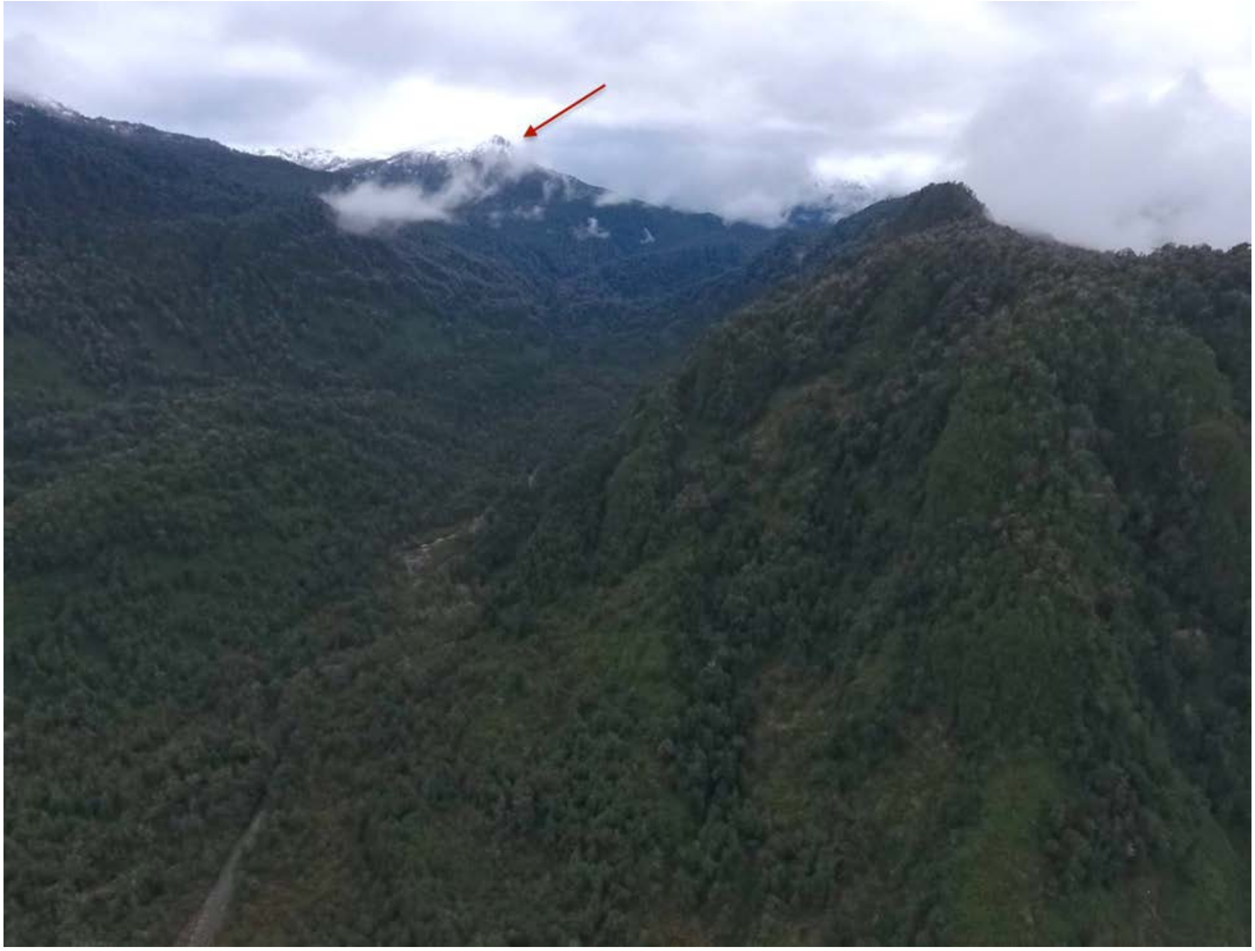

Drone photograph looking north from the Quitralco Fiord of the main youngest cone of the VMG taken in October 2018 during the CIMAR24 research investigation. Red arrow indicates the youngest cone shown in previous photos that formed within the caldera. Stream in the photo flows <4 km from the VMG to the edge of Quitralco Fiord where samples were collected (see following photo). Drone photograph by De Pascale.

Fig. S14.

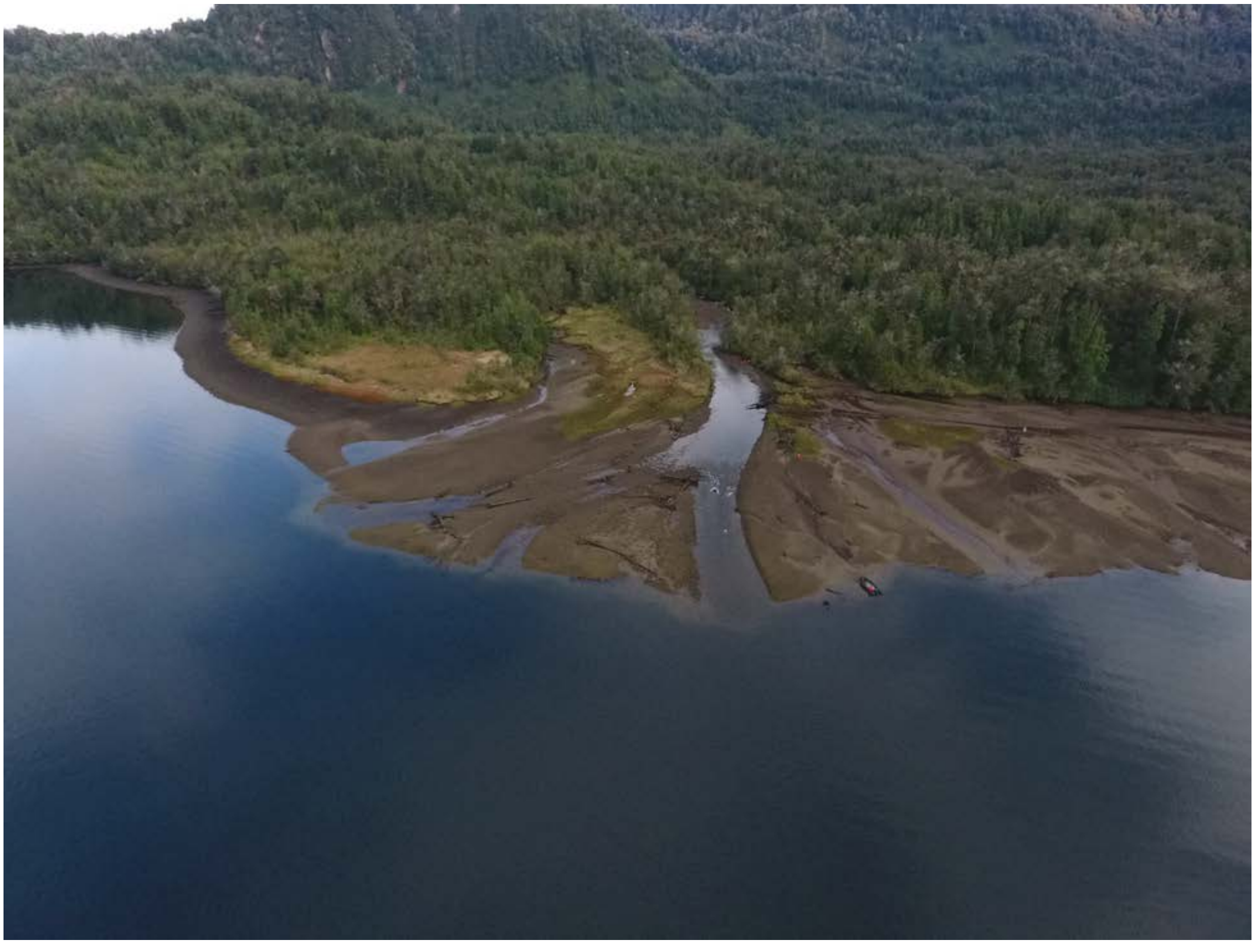

Drone photograph of the creek (Volcan Mate Grande Creek – informal name) draining the VMG caldera (<4 km from the youngest VMG cone) where it is forming a modern progradational delta in the Quitralco Fiord immediately south of VMG. Note the zodiac boat and researchers in orange survival suits sampling the rocks (lavas and tuffs) derived from the VMG. Drone photograph by De Pascale.

Fig. S15.

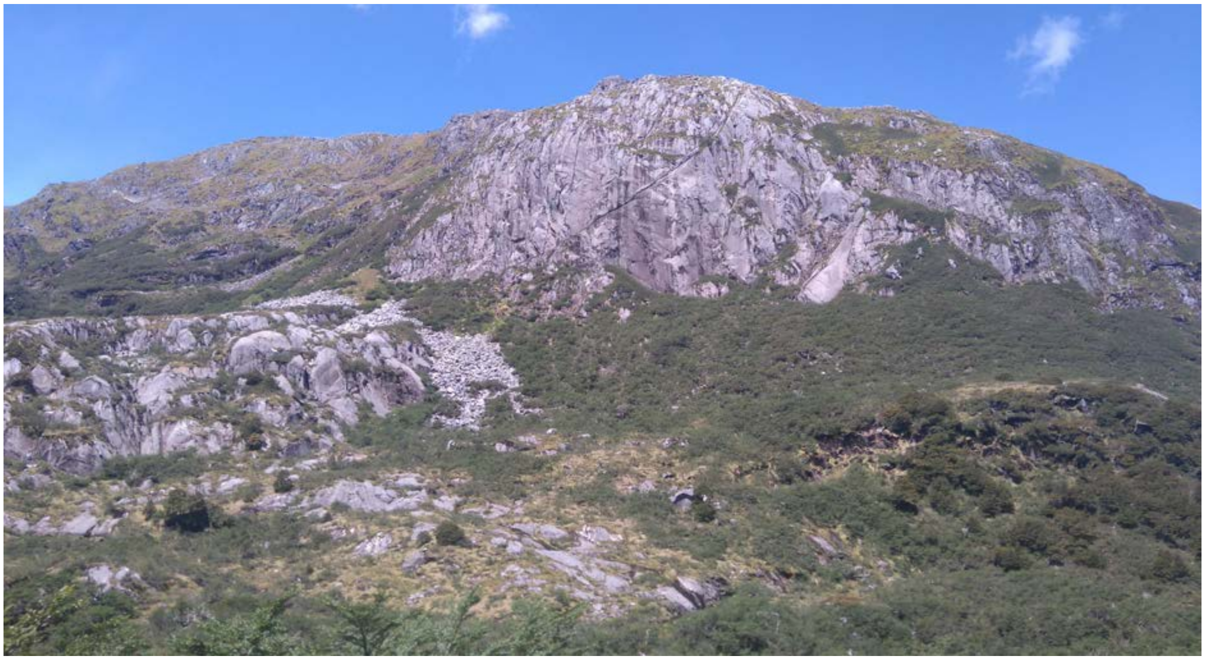

Field photograph in the Huemules Cirque (looking south) showing the nature of the Miocene Patagonian Batholith (Mg). Note the light (felsic) colours common in the Patagonian Batholith in contrast with dark (mafic) colours in Helicopter photographs of the volcanic rocks found in the Volcan Mate Grande in the above photos. Note the absence of evidence for hydrothermal alteration here and smooth glacially polished nature of this area due to the strength of these intrusive rocks. This site is ~30 km south-southwest of the Volcan Mate Grande. Photo by De Pascale.

Fig. S16.

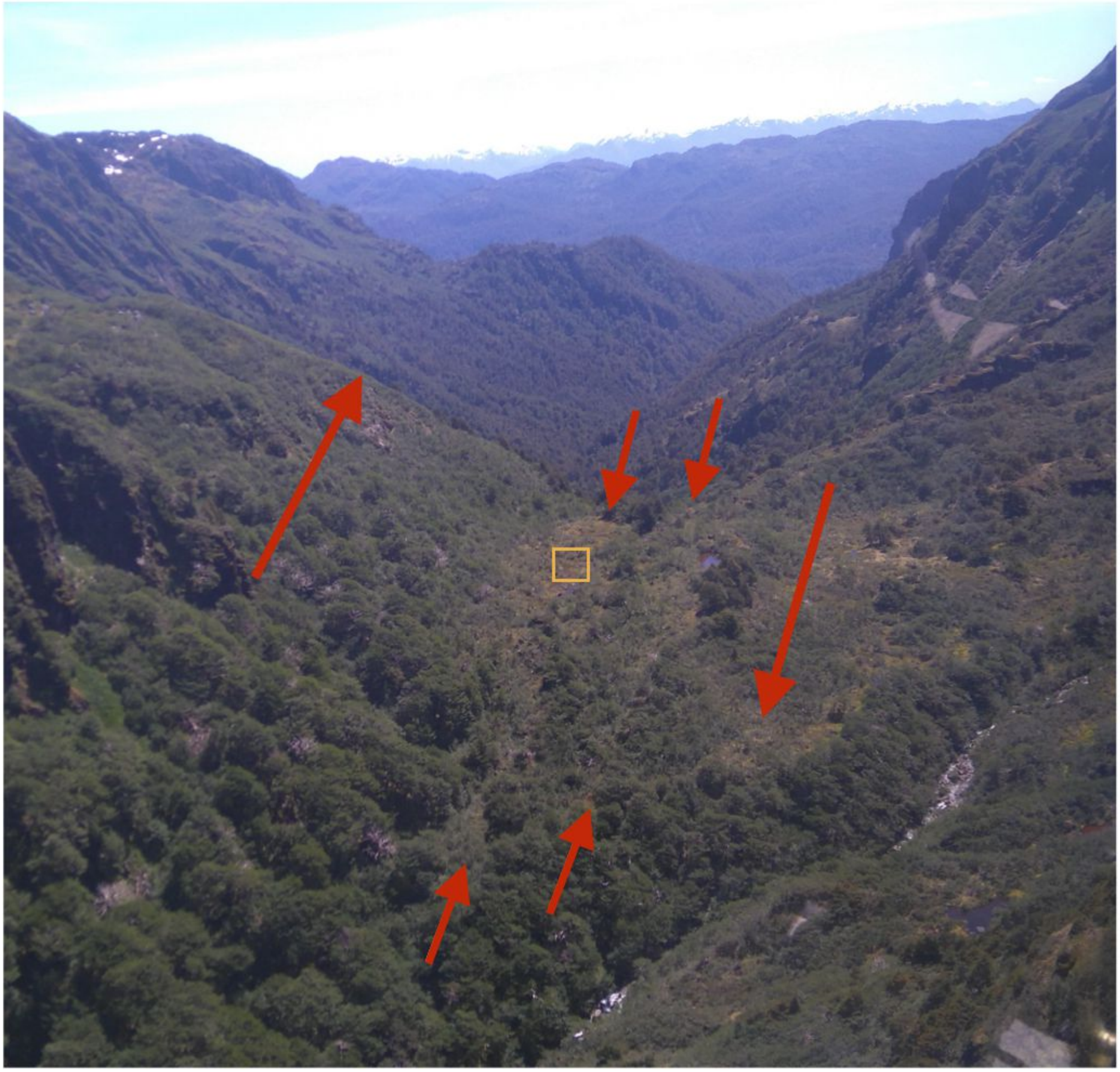

Helicopter photograph looking north along-strike of the LOFZ at the Huemules Cirque. Small red arrows are showing two distinct lineaments coincident with the main trace of the LOFZ. Large red arrows showing sense of slip (with left side moving northwards). Orange box is area of photo below showing sag ponds in the field coincident the the lineaments found in this photo. Photo by De Pascale.

Fig. S17.

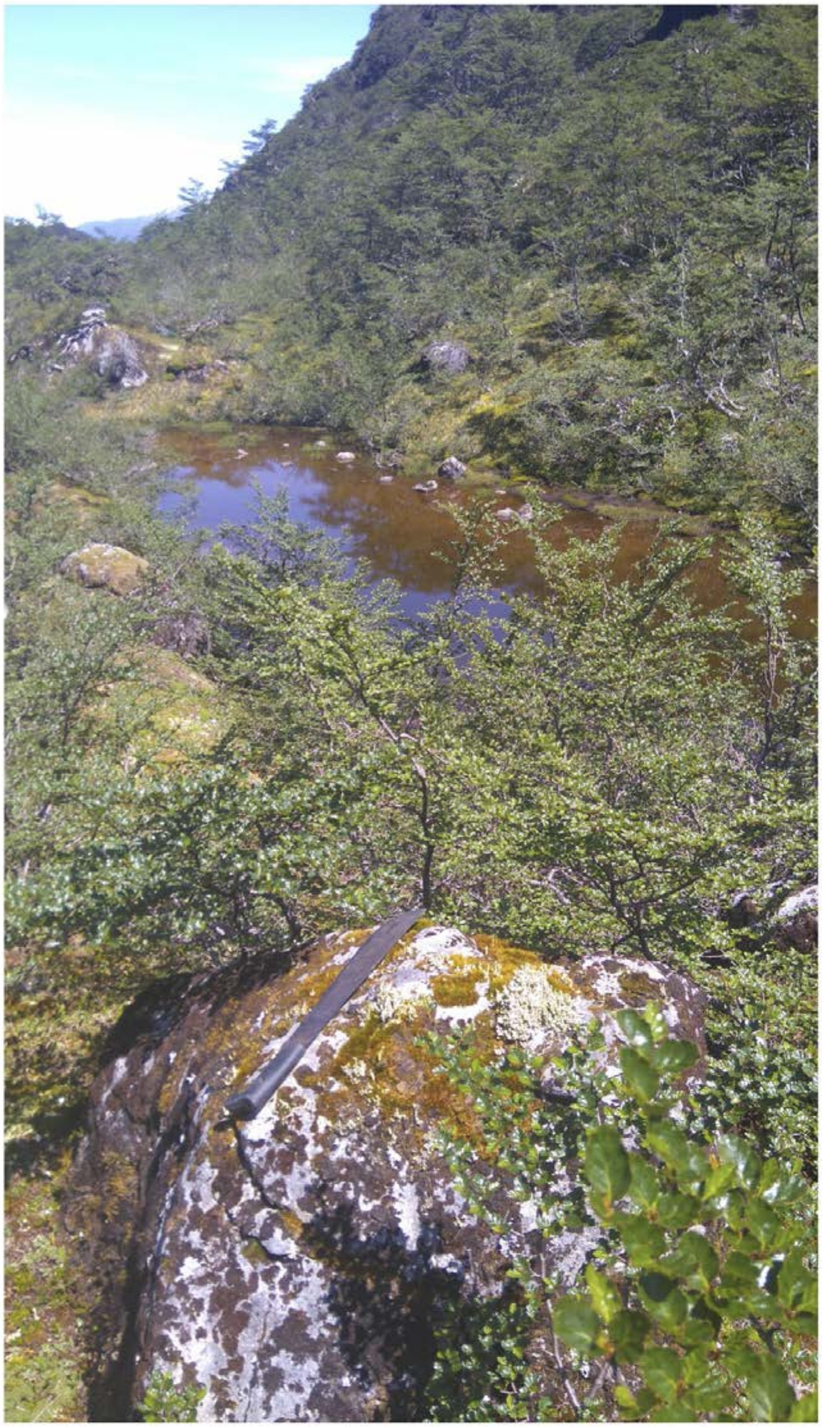

Sag ponds found along-strike of the LOFZ. Field photo showing boulders at the top of this moraine in which the lineaments along the LOFZ formed. Machete for scale. Photo by De Pascale.

Fig. S18.

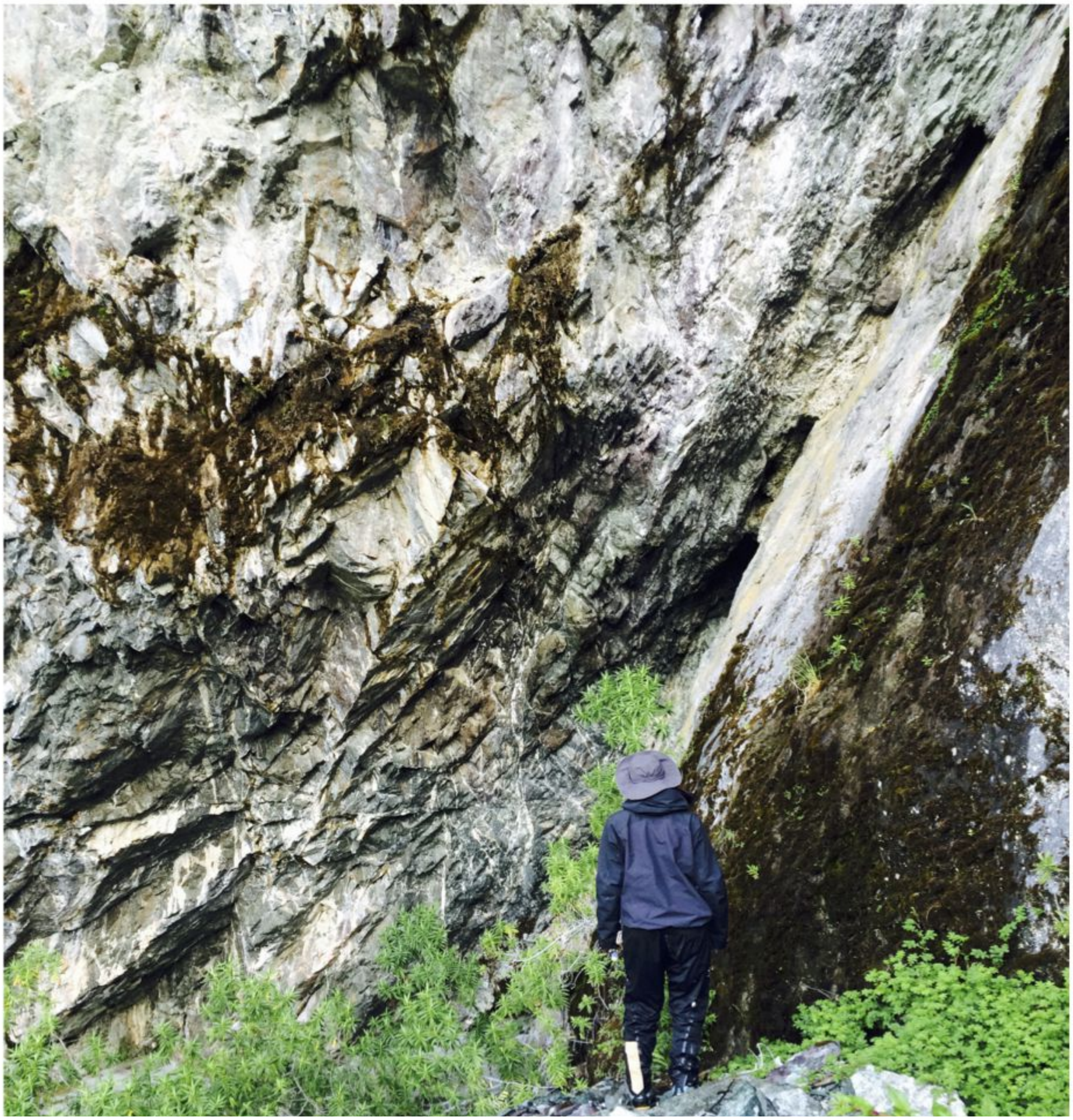

Field photograph of the west-northwest dipping (018/75 NW) principal slip zone (PSZ) or fault core of the LOFZ (composed of cataclasite and gouge) coincident with the offset observations in below photographs that we discovered in December 2015. Note the clear fault plane to the right of the person (for scale) and how the fault rocks are increasing finer as they approach the principle slip zone (upper right corner of photo). Also note the sub-vertical calcite and fault gouge-filled veins left of the person. This is where samples were acquired for the fluid inclusion work outlined below. Above the person is breccia (i.e. brittle deformation fault rocks) throughout. To our knowledge this is the best LOFZ fault core exposed from any documented site along it's ~1200 km length. Photo – December 2016 by De Pascale.

Fig. S19.

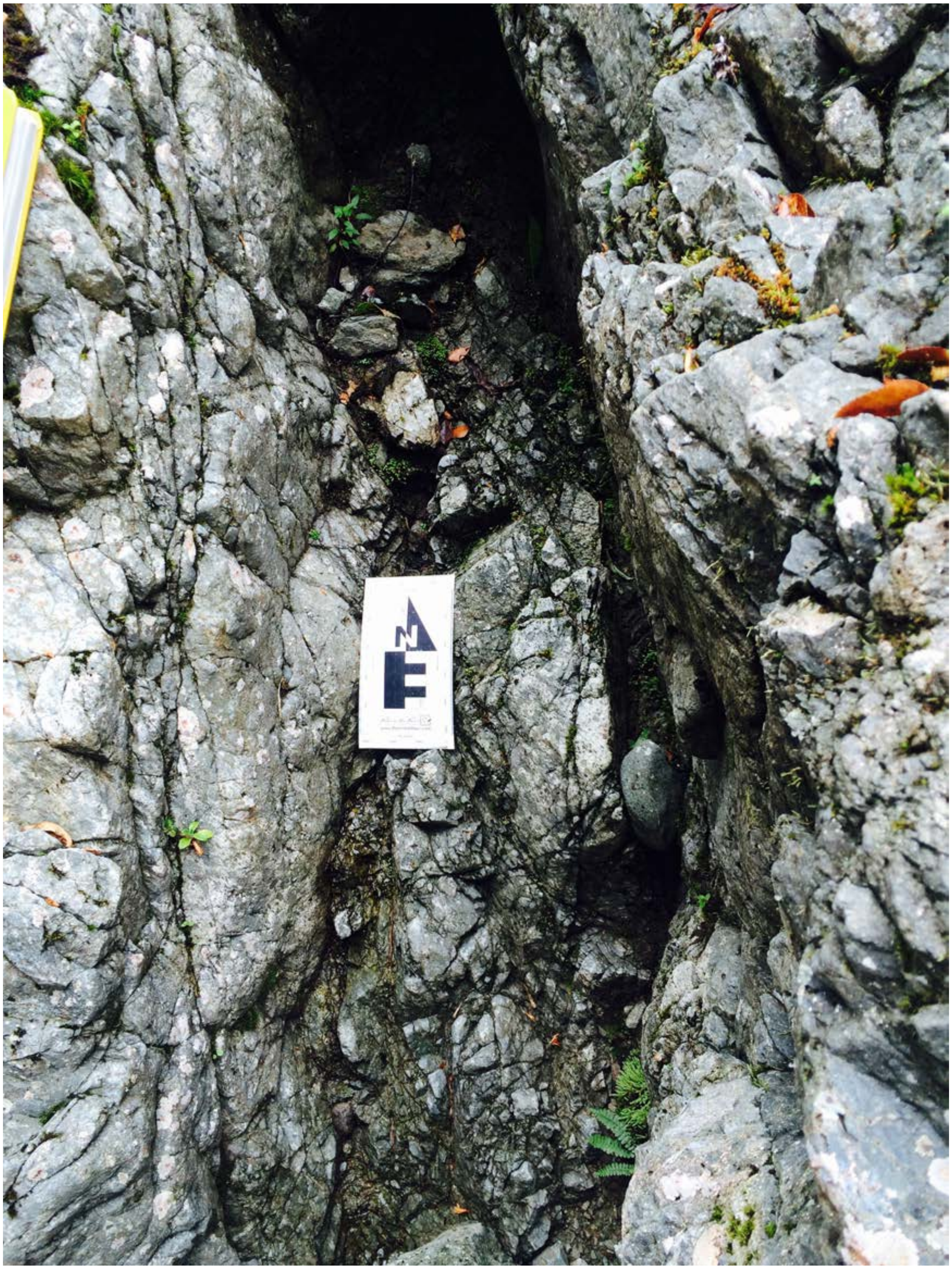

Field photographs of fault rocks along the LOFZ coincident with short (<20 m) dextral displacements near the Laguna San Rafael. Note the breccia and cataclasite (right of the scale bar) here. Thin lenses of plastic fault gouge were also found here. Photo by De Pascale.

Fig. S20.

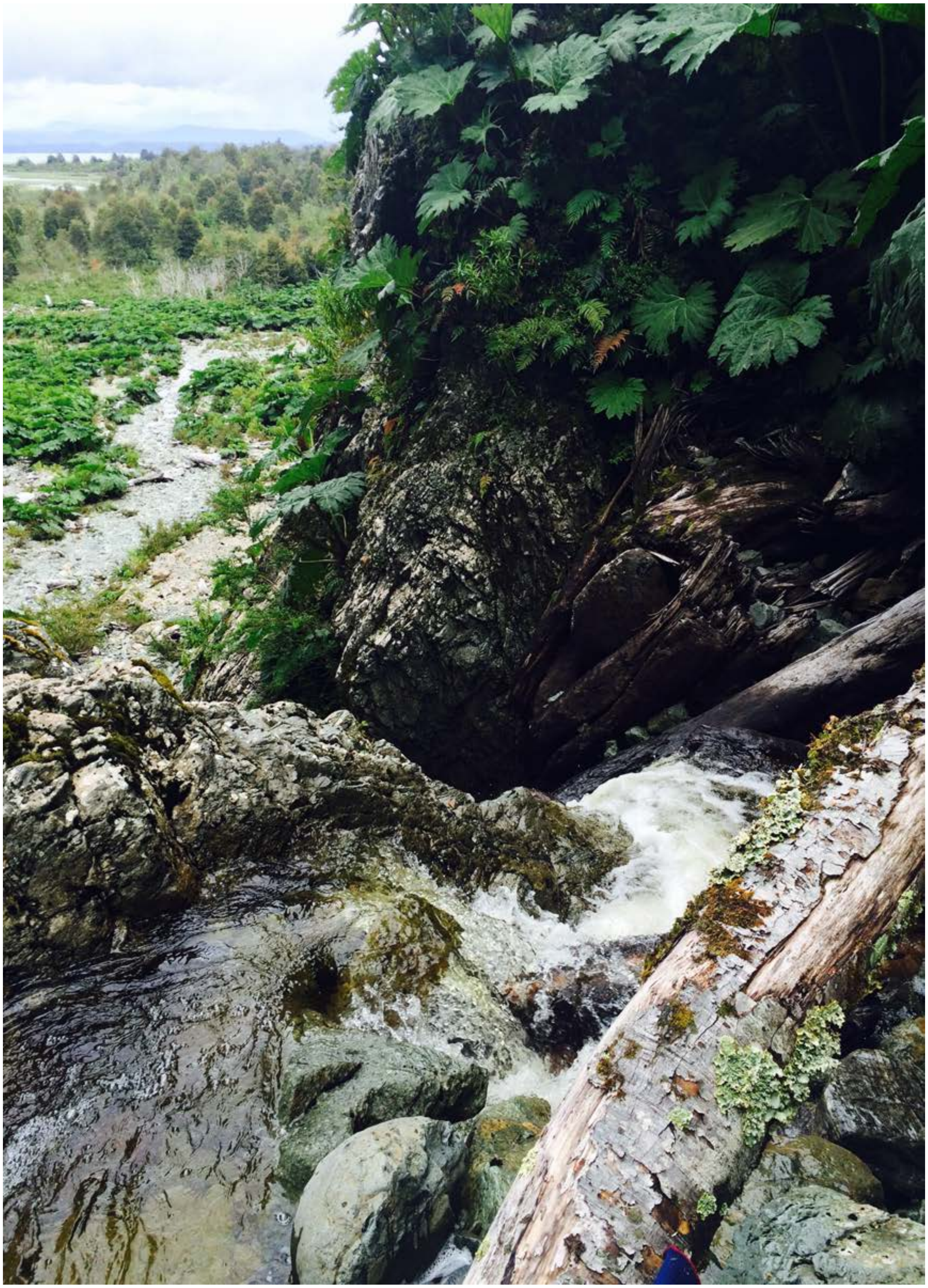

Field photograph showing the dextral displacement of the thalweg of a stream coincident with fault rocks shown in the above two photos along the LOFZ. Photo by De Pascale.

Fig. S21.

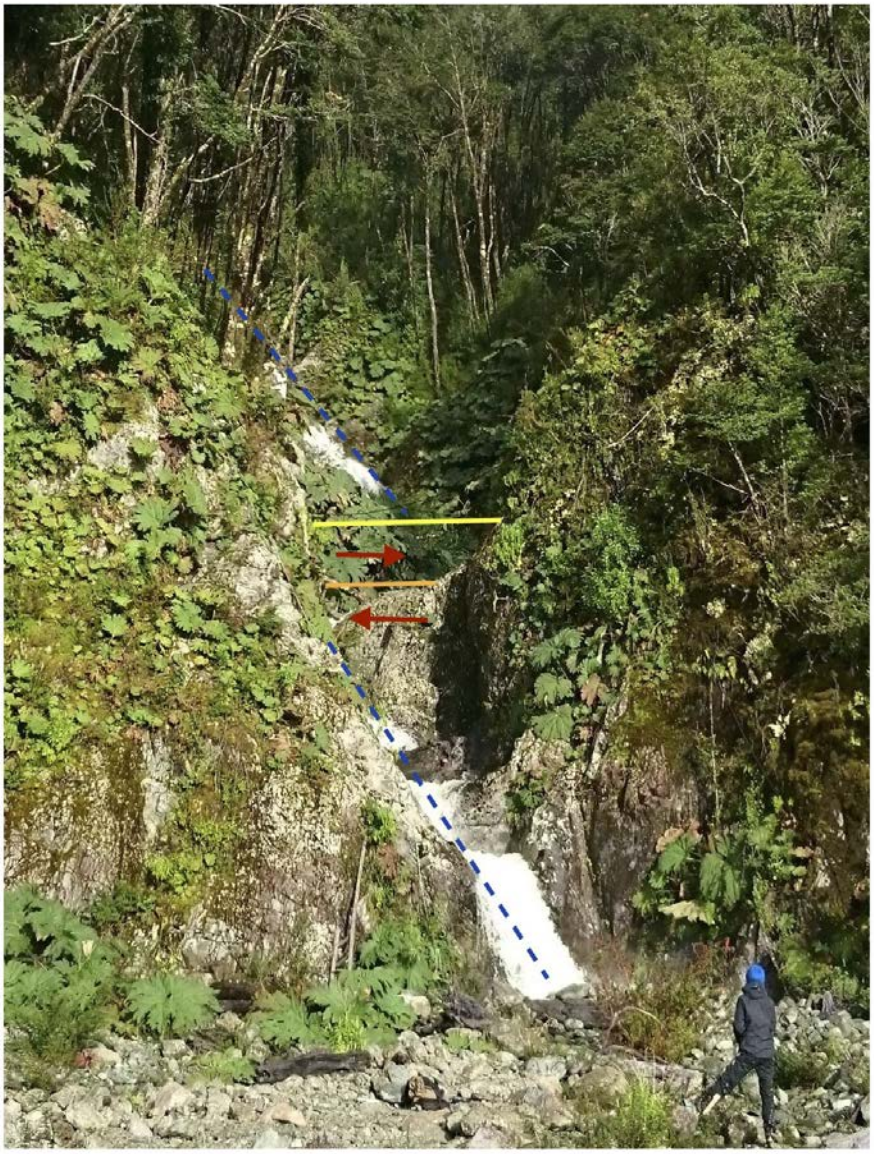

Field photograph looking Southeast that shows a creek exiting the range front with a waterfall at the LOFZ north of the Laguna San Rafael. The site was visited to validate mapping of the clear geomorphic trace of the fault north of the Ofqui Peninsula. The steeply dipping (76ºNW) brecciated zone's strike is coincident with an ~8 dextral offset of the creek thalweg (orange line) and a larger 17.8 m dextral offset (yellow line) of the creek's canyon (thalweg shown - dashed blue line & dextrally offset), which strongly suggests recent dextral-reverse motion along the LOFZ fault rocks. Here we discovered two outcrops of the LOFZ fault core (shown in the previous photos) that show clear brittle deformation with subvertical layers of breccia, cataclasite, and calcite gouge (i.e. fault rocks) coincident with the geomorphic fault trace and short geomorphic displacements. Sense of slip shown with red arrows. Note the offsets are not clearly scaled and are shown for indicative purposes only. Although some dip slip is likely here, the waterfall height is likely influenced by glacial erosion from the San Rafael Glacier within the past couple of hundred years. Person for scale. Photo – April 2015 by De Pascale.

Fig. S22.

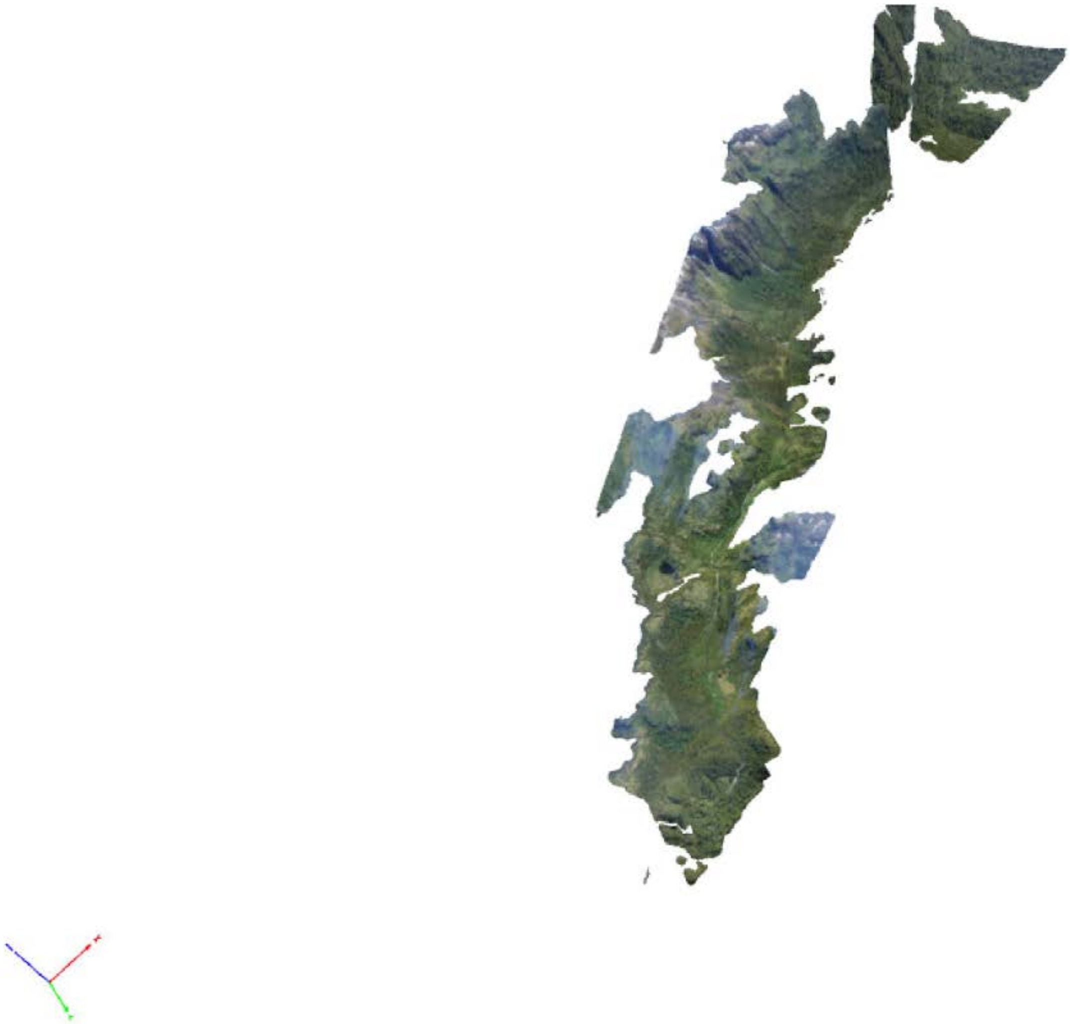

Screen capture of the 3D SfM model of the Huemules Cirque area with full coverage of the model. Note areas with white were not covered with our field photogrammetry survey and thus there is no data there. This file is available to be viewed as Data S1 – which is a 3D pdf model that can be viewed with Adobe Acrobat software as a 3D file. Software used to develop this model was Agisoft Standard Photoscan Pro 1.3.2 (2018)[2]. Accessed through https://www.agisoft.com.

Fig. S23.

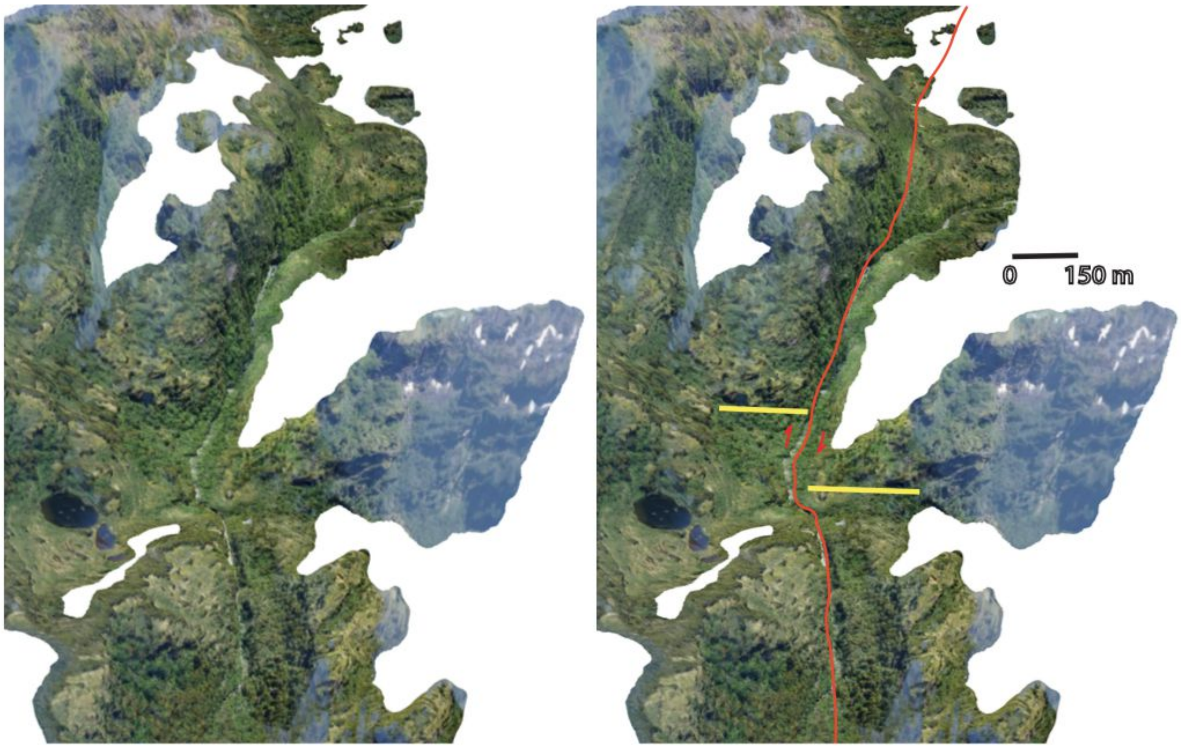

Figure derived from the Structure from Motion (SfM, Data S1) 3D model along the LOFZ with interpretations on right. Looking northward along the LOFZ with the dextrally displaced cliff (yellow line) that is mention in Table 1 in the main text. We feel that this represents younger than Younger Dryas deformation. Sag ponds shown in the above figures are in the area adjacent and left of the scale bar. Full model can be found in Data S1 – which is a 3D pdf model that can be viewed with Adobe Acrobat software as a 3D file. Software used to develop this model was Agisoft Standard Photoscan Pro 1.3.2 (2018)[2]. Accessed through https://www.agisoft.com.

Table S1.

LOFZ 4	3.6	Between the Rio Cupquelan and the Rio De Los Huemules.	Concealed by the lahar plain of the Hudson Volcano which flows down the Huemules river.	Concealed by the lahar plain of the Hudson Volcano which flows down the Cupquelan.
LOFZ 5	19.5	From just north of Huemules River (lahar plain of the Hudson Volcano), running through the Huemules range north to the southern side of the Quitralco Fiord.	Submarine at the Quitralco fiord and immediately north of the fiord is buried by Holocene volcanics of the Mate Grande Volcano we discovered during mapping of the fault in our study.	Lost in the high topography of the Southern Huemules range just north of the Huemules river.
LOFZ 8	5.3	From the Northern side of the Quitralco Fiord cutting through the MGV Caldera and youngest cone to just outside of the Caldera rim.	Very young valley (alluvium).	Shallow submarine (nearshore).
LOFZ 7	13.5	From north of Mate Grande Volcano north to the Aysen Fiord	Submarine along the Aysen Fiord.	Concealed by the Mate Grande Volcano.
LOFZ 8	8	From Aysen Fiord north into young volcanic deposits and cones from the Maca and Cay Volcanos in the Rio Cuervo	Concealed by volcanic deposits and cones from the Maca and Cay Volcanos.	Submarine in the Aysen Fiord.
LOFZ 9	12.9	From just north of the Cay Volcano to the Southern edge of the Canal Puyuhapi.	Submarine in the Canal Puyuhapi.	Concealed by Cay Volcano deposits.
	103.9	**Total length mapped trace (km)**	**Notes**	
	400	**Total length of section from Golfo de Pena to Aysen Fiord**	*Additional 100 km south in Golfo de Pena.*	
	25.98%	**Proportion of LOFZ mappable**		

Breakdown of fault mapping of the main trace of the LOFZ and reasons and where the fault trace cannot be accurately mapped.

Table S2.

**Maximum Area (km2)**	132 km2	Google Earth
**Activity Evidence**	Post-Last Glacial Maximum (LGM), i.e. post 17.3 ka. Likely Holocene (last 11 ka) based on cone and caldera morphology, and LOFZ fault trace cutting the Quaternary volcanic landslide (and youngest cone).	Helicopter reconnaissance, Google Earth
**Date of last eruption**	Holocene but exact data unknown but perhaps the source of the unknown eruptions at 5200 yr BP and/or 2000 yr BP based on the work by Wils et al., 2018	Wils et al., 2018

Baseline characteristics of the Volcano Mate Grande discovered during this investigation.

Data S1. Structure from Motion (SfM) model from the Huemules Cirque

This 3D SfM model was developed taking overlapping photographs flying along the LOFZ by helicopter. It can be viewed in Adobe Acrobat software as a 3D pdf file and provides a 3D model along the LOFZ crossing the Huemules Cirque.

Data S2. Excel file with the geochemical data from the Mate Grande Volcano

This is the entire geochemical dataset for the Mate Grande Volcano that was used for the analysis presented in Figure 2 analysis of the main paper. The lab data was from ALS Patagonia S.A. And was for 4 acid multi-element ICP-MS with rare earth elements. Additionally XRF analysis was undertaken.

Supplemental References

1. Google Earth version 7.1.5.1557. Accessed through https://www.google.com/earth/.

2. Agisoft Standard Photoscan Pro 1.3.2 (2018). Accessed through https://www.agisoft.com.
